# Stem cell-derived extracellular vesicles -mediated bone regeneration: mechanisms, targeted delivery, and clinical perspectives in promoting angiogenesis

**DOI:** 10.3389/fbioe.2026.1784567

**Published:** 2026-04-10

**Authors:** Yang Shu, Zhiqiang Luo, Zhi Tang, Wei Li, Fei Sun, Ting Dong, Cheng Zhang, Xiaobin Luo, Zhixiong Luo, Xijuan Liu, Wenxing Liang, Min Zhou, Xiaohui Zhang, Xiangqi Qin, Wenjie Su, Hui Xiong

**Affiliations:** 1 Department of Orthopedics, The First Affiliated Hospital of Hunan University of Chinese Medicine, Changsha, Hunan, China; 2 Department of Graduate School, Hunan University of Chinese Medicine, Changsha, Hunan, China; 3 Department of Orthopedics, Xiangtan Municipal Hospital of Traditional Chinese Medicine, Xiangtan, Hunan, China; 4 Department of Geriatrics, Xiangtan Municipal Hospital of Traditional Chinese Medicine, Xiangtan, Hunan, China; 5 Department of Orthopedics, Xupu Chengnan Hospital, Xupu, Hunan, China; 6 Department of Orthopedics, Longhui Hospital of Traditional Chinese Medicine, Shaoyang, Hunan, China; 7 Department of Geriatrics, The First People’s Hospital of Changde, Changde, Hunan, China; 8 Department of Geriatrics, People’s Hospital of Mengzi, Mengzi, Yunnan, China; 9 Department of Respiratory Medicine, Xiangtan Municipal Hospital of Traditional Chinese Medicine, Xiangtan, Hunan, China; 10 The First Clinical Medical College, Heilongjiang University of Chinese Medicine, Harbin, Heilongjiang, China; 11 Department of Orthopedics, Shaoyang Hospital of Traditional Chinese Medicine, Shaoyang, Hunan, China

**Keywords:** angiogenesis, biomaterial delivery, bone regeneration, clinical translation, extracellular vesicles, mesenchymal stem/stromal cells, osteogenesis

## Abstract

Angiogenesis is indispensable for bone regeneration and tightly coupled to osteogenesis, yet inadequate vascularization remains a common cause of repair failure in critical-sized defects, compromised fractures, and osteochondral lesions. Conventional growth-factor delivery and cell transplantation rarely reproduce the spatiotemporal vascular program required for functional healing, motivating the search for controllable and translatable alternatives. Mesenchymal stem/stromal cell (MSC)–derived extracellular vesicles (EVs) have emerged as a modular, cell-free modality that packages regenerative cues into lipid-bilayer nanoparticles capable of coordinating endothelial activation, immune reprogramming, and osteogenic differentiation. In this review, we (i) delineate phase-resolved angiogenic events across inflammation, callus formation, mineralization, and remodeling; (ii) synthesize mechanistic evidence showing how EV cargos—including microRNAs (miRNAs), proteins, and lipids—promote sprouting, vessel stabilization, and angiogenesis–osteogenesis coupling across models of fractures, segmental/critical-sized defects, osteonecrosis, and alveolar/osteochondral repair; and (iii) critically appraise engineering and delivery strategies (preconditioning, cargo loading, surface functionalization, and biomaterial depots) that enhance lesion exposure, local retention, and sustained bioactivity. To bridge proof-of-concept and a regulatory-ready therapeutic product, we further summarize manufacturing scale-up, quality control beyond minimal EV identity markers, mechanism-anchored potency assays, dosing metrics, biodistribution, and long-term safety considerations, and we highlight the nascent but evolving clinical landscape in bone and joint disorders. Collectively, this review provides a practical roadmap for developing reproducible EV therapeutics that enable vascularized bone regeneration.

## 1 Introduction

Bone regeneration—particularly the repair of critical-sized segmental defects—remains a major clinical challenge. Owing to inadequate vascular supply, segmental defects rarely heal spontaneously and frequently culminate in delayed union or non-union (Schemitsch, 2017). In both bone grafting and bone tissue engineering, insufficient neovascularization within the injured region is widely recognized as a key barrier to robust regeneration and graft integration (Menger et al., 2022). Newly formed vessels not only deliver oxygen and nutrients to the regenerating tissue but also provide indispensable conduits for the recruitment and migration of osteoblast-lineage and other reparative cells. Accordingly, angiogenesis is a decisive determinant of bone regeneration and a prerequisite for the clinical translation of bone tissue engineering strategies.

To improve vascularized bone regeneration, numerous strategies have been developed, including pro-angiogenic growth-factor delivery, cell-based transplantation, and tissue-engineered scaffolds designed to enhance vessel ingrowth or provide pre-vascularized networks. However, several limitations remain unresolved (Simunovic and Finkenzeller, 2021). Soluble growth factors often have rapid diffusion and short *in vivo* half-lives, resulting in burst release and heterogeneous exposure; moreover, their therapeutic window is narrow and supraphysiologic dosing can induce aberrant or leaky vasculature and off-target effects (García and García, 2016). Pre-vascularized constructs and complex scaffold designs can accelerate early perfusion but are technically demanding, difficult to standardize, and do not always achieve rapid, stable anastomosis with host vasculature in large defects. Meanwhile, targeted delivery systems and nanoparticles can improve local retention, yet bone-homing efficiency, immune clearance, payload stability, and limited ability to deliver coordinated multi-signal instruction across healing phases continue to constrain predictable clinical translation (Niu et al., 2025).

In recent years, stem cell–based approaches have attracted intense interest for their regenerative potential. Among them, mesenchymal stem/stromal cells (MSCs) have been extensively studied and consistently shown to facilitate bone repair (Grayson et al., 2015). However, conventional MSC transplantation faces notable limitations, including immunogenicity, potential tumorigenic risk, and poor *in vivo* survival (Chen W. et al., 2023). Accumulating evidence now indicates that the therapeutic efficacy of MSCs is mediated primarily not by direct differentiation but by paracrine mechanisms. In particular, MSC-secreted extracellular vesicles are increasingly viewed as central effectors of MSC paracrine signaling (Spees et al., 2016). Notably, EV-mediated paracrine signaling is a two-party communication that requires defining both an EV-producing “sender” cell and a signal-decoding “receiver” cell. In bone repair, MSCs (or other engineered producer cells) serve as key senders, whereas endothelial cells, immune cells (e.g., macrophages), osteoprogenitors/osteoblast-lineage cells, and chondrocyte-lineage cells represent major receivers that interpret EV cargos via uptake and ligand–receptor interactions (Wang et al., 2024).

MSC-derived extracellular vesicles are enriched in bioactive cargos such as mRNAs, microRNAs, and proteins. For clarity, we distinguish transcript-level (gene) readouts from protein-level readouts: gene symbols (e.g., *VEGFA*) are used when describing mRNA/gene expression, whereas protein nomenclature (e.g., *VEGF-A*) is used when describing protein cargos or protein-level changes. These cargos can be efficiently transferred between cells, reprogramming gene expression and biological functions in recipient cells and thereby orchestrating tissue repair and regeneration (Kim et al., 2024). Compared with cell-based therapies, extracellular vesicles -based, cell-free approaches offer several advantages, including low immunogenicity, improved storage and transportability, and avoidance of uncontrolled cell proliferation or differentiation. Consequently, MSC-derived extracellular vesicles have emerged as a promising alternative to traditional stem cell therapies and show considerable potential in regenerative medicine, particularly in bone regeneration (Tang et al., 2024).

Notably, MSC-derived extracellular vesicles exhibit pronounced pro-angiogenic activity and have become a major focus of current research. Multiple studies demonstrate that delivering MSC-derived extracellular vesicles to animal models of bone injury can markedly enhance angiogenesis at the defect site, thereby accelerating bone repair (Zhu et al., 2019; Takeuchi et al., 2019). Nevertheless, the precise molecular mechanisms underlying extracellular vesicles -driven angiogenesis remain incompletely defined. How distinct exosomal cargos act in concert, and how downstream effector cells interpret these signals, require systematic clarification. Elucidating these mechanisms will not only improve the efficacy and predictability of extracellular vesicles-based therapies but also provide a mechanistic foundation for precision intervention strategies.

Against this backdrop, this review aims to comprehensively summarize the molecular mechanisms by which stem cell–derived extracellular vesicles promote angiogenesis during bone regeneration, highlight strategies for targeted delivery, and discuss translational pathways toward clinical use. We first outline stage-specific features of angiogenesis during bone repair. We then synthesize current evidence—primarily from MSC-derived extracellular vesicles—across diverse bone defect models and systematically describe the molecular programs that govern exosome-mediated vascular formation. Next, we discuss how biomaterial carriers and engineering modifications can be leveraged to optimize targeted delivery and local retention of extracellular vesicles within bone defects, thereby enhancing both angiogenic and osteogenic outcomes. Finally, we address opportunities and challenges in translating stem cell–derived exosomes from bench to bedside, with emphasis on scalable manufacturing, safety evaluation, biodistribution, and the evolving landscape of preclinical and clinical progress. We anticipate that this review will provide a structured reference and inspire future directions for extracellular vesicles-driven angiogenesis and bone regeneration.

## 2 Stage-specific angiogenesis during bone repair

**FIGURE 1:**
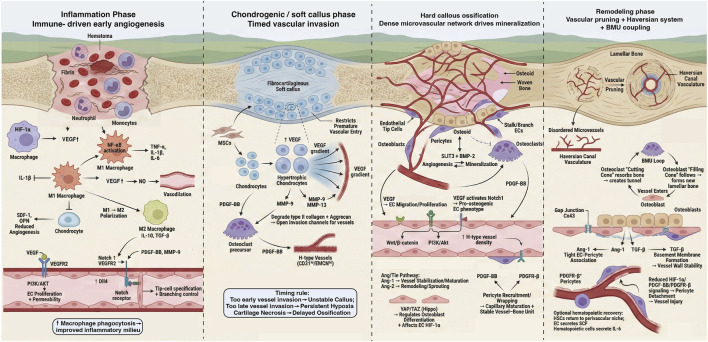
Stage-specific angiogenesis during bone fracture repair. Schematic summary of vascular events and key cellular interactions across four sequential phases of long-bone fracture healing (left to right). (1) Inflammation/hematoma phase: immune-cell infiltration (neutrophils, monocytes/macrophages) and hypoxia induce pro-angiogenic cytokines and VEGF, increasing endothelial sprouting and permeability via VEGFR2–PI3K/AKT and Notch-mediated tip/stalk selection. (2) Chondrogenic/soft callus phase: hypertrophic chondrocytes and MSCs establish a VEGF gradient while matrix metalloproteinases open invasion channels for timed vascular entry; premature invasion can destabilize callus. (3) Hard callus ossification: a dense microvascular network supports mineralization; coupling cues (e.g., PDGF-BB–PDGFRβ pericyte recruitment, Ang/Tie signaling, SLIT3 and BMP-2) coordinate angiogenesis with osteoblast activity and H-type vessel enrichment (CD31hi/EMCNhi). (4) Remodeling phase: vascular pruning, Haversian canal formation and BMU coupling between osteoclast “cutting cones” and osteoblast “filling cones.” Created by the authors based on representative studies (Einhorn and Gerstenfeld, 2015; Street et al., 2002; Hu and Olsen, 2016; Kusumbe et al., 2014). Abbreviations: Ang, angiopoietin; BMU, basic multicellular unit; BMP, bone morphogenetic protein; Cx43, connexin-43; DLL4, Delta-like ligand 4; EC, endothelial cell; EMCN, endomucin; HIF-1α, hypoxia-inducible factor-1α; HSC, hematopoietic stem cell; IL, interleukin; MMP, matrix metalloproteinase; MSC, mesenchymal stromal/stem cell; NF-κB, nuclear factor-κB; NO, nitric oxide; OPN, osteopontin; PDGF, platelet-derived growth factor; PDGFRβ, platelet-derived growth factor receptor-β; PI3K, phosphoinositide 3-kinase; SDF-1, stromal cell-derived factor 1; SLIT3, slit guidance ligand 3; TAZ, transcriptional co-activator with PDZ-binding motif; TGF-β, transforming growth factor-β; TNF-α, tumor necrosis factor-α; VEGF, vascular endothelial growth factor; VEGFR2, VEGF receptor 2; Wnt, Wingless/Int; αSMA, alpha-smooth muscle actin.

Bone regeneration after fracture proceeds through the inflammatory phase, the cartilaginous (soft callus) phase, the hard callus (mineralization) phase, and the remodeling phase. Angiogenesis is indispensable throughout this process: beyond supplying oxygen and nutrients, neovessels couple tightly with osteogenesis through angiocrine signaling (Grosso et al., 2017; Zhu et al., 2020). In this section, we summarize angiogenic features across stages, key regulators, major cellular players and their crosstalk, and emerging intervention strategies that modulate vascularization (Figure 1).

Recent single-cell and single-nucleus sequencing together with spatial transcriptomic analyses have refined the cellular atlas of bone regeneration, highlighting that the umbrella term “MSC” encompasses heterogeneous stromal/progenitor subsets and that distinct fibroblast populations and progenitor-derived chondrocyte subtypes coexist and evolve across repair phases (Wang H. et al., 2025). These cell states differ in receptor–ligand landscapes and therefore respond differently to stage-specific cues, including EV cargos. Accordingly, throughout this review we specify the donor EV source (sender) and the principal recipient populations (receivers) when describing mechanisms of action, and we relate EV effects to the evolving cellular microenvironment across repair stages (Perrin et al., 2024).

Although “VEGF” is often used as a generic term, the VEGF family comprises multiple ligands (VEGF-A, VEGF-B, VEGF-C, VEGF-D, and placenta growth factor, PlGF) with distinct receptor preferences and roles in vascular biology (Hu and Olsen, 2016). Unless otherwise specified, VEGF in this review refers to VEGF-A (encoded by *VEGFA*), which predominantly activates VEGFR2 to drive blood-vessel sprouting and vascular permeability and is the best-characterized VEGF isoform in bone repair. VEGF-B and PlGF primarily bind VEGFR1 and can modulate endothelial survival and inflammatory cell recruitment, thereby shaping the reparative microenvironment (Albonici et al., 2019). VEGF-C and VEGF-D preferentially signal through VEGFR3 and regulate lymphangiogenesis, which may contribute to edema clearance and inflammation resolution during fracture healing (Zheng et al., 2025). Accordingly, we specify VEGF subtypes at relevant points below to better match stage-specific biological processes with the corresponding VEGF signaling axis.

### 2.1 Angiogenesis in the inflammatory phase

Within hours to days after fracture, the injury enters the inflammatory phase. Immune cells—including neutrophils and monocytes/macrophages—are rapidly recruited to the lesion, forming a hematoma and releasing inflammatory mediators that initiate debris clearance and the repair program. The resulting hypoxic microenvironment stabilizes hypoxia-inducible factor 1α (HIF-1α), triggering an angiogenic cascade (Stucker et al., 2020; Rui et al., 2025).

During this phase, immune responses are intimately coupled to angiogenesis; an appropriately tuned inflammatory milieu is necessary to support early vascular sprouting (Baht et al., 2018). Macrophages are central regulators in this context (Capobianco et al., 2024). Early infiltrating M1-like macrophages secrete tumor necrosis factor-α (TNF-α), interleukin-1β (IL-1β), interleukin-6 (IL-6), contributing to osteoclast activation and debris clearance, while also producing VEGF-A to initiate endothelial sprouting and generating nitric oxide (NO) via iNOS to promote vasodilation (Aguilar et al., 2020). As inflammation resolves, macrophages progressively shift toward an M2-like phenotype, secreting anti-inflammatory mediators (e.g., IL-10 and TGF-β) to prevent tissue damage from excessive inflammation, and producing PDGF-BB and MMP-9 to promote vessel stabilization and remodeling (Chen S. et al., 2023; Gou et al., 2024). In turn, endothelial cells secrete chemokines such as CXCL12 to recruit fibroblasts and osteoprogenitors to participate in repair (Zhu et al., 2020; Hostler et al., 2024).

Importantly, the dynamic balance between M1-and M2-like macrophage states is critical for physiological angiogenesis. Either excessive or insufficient inflammation can disrupt this balance and compromise vascular formation (Muire et al., 2020). When inflammation is overly intense or prolonged—as in rheumatoid arthritis or diabetes—pro-angiogenic programs are often blunted and angiogenesis is impaired (Wang et al., 2021). Chronic hyperinflammation characterized by persistently high IL-1β and TNF-α is particularly inhibitory to angiogenic signaling and is associated with an increased risk of non-union (Lim et al., 2017). Conversely, inadequate early inflammation—for example, due to premature or excessive use of broad anti-inflammatory drugs—may also suppress vascular invasion and impede callus formation.

Key pathways in this stage include nuclear factor κB (NF-κB) and HIF-1α. Activation of NF-κB promotes sustained production of pro-inflammatory cytokines (e.g., IL-1β and TNF-α), which—when excessive—can dampen angiogenic signaling. For instance, IL-1β has been reported to downregulate chondrocyte secretion of SDF-1 and osteopontin (OPN), contributing to insufficient angiogenesis (Wang et al., 2021). Under hypoxia, stabilized HIF-1α induces VEGF expression; VEGF-A then activates PI3K/AKT signaling through VEGFR2 to promote endothelial proliferation and vascular permeability (Wang X. et al., 2020). Notch signaling also contributes early: hypoxia and inflammation can upregulate endothelial Delta-like ligand 4 (Dll4), which engages Notch receptors on neighboring endothelial cells to specify “tip cell” differentiation and regulate vascular branching (Diez et al., 2007; Blanco and Gerhardt, 2013). Notably, Notch signaling in bone endothelium appears context-specific: Notch upregulation can enhance VEGFR2 expression and promote neovascular growth in bone, differing from the anti-angiogenic role often described in other tissues (Ramasamy et al., 2016). In addition, Wnt/β-catenin signaling primarily supports MSC recruitment and survival during early repair; however, premature or excessive activation may disrupt the subsequent cartilaginous phase, suggesting that a moderate level of early Wnt activity is more favorable for coordinated healing (Chen et al., 2007).

Neurogenic factors are increasingly appreciated as contributors to early repair. Nerve fibers can enter the injury region alongside neovessels (Qin et al., 2022). And neurotrophic factors (e.g., NT-3) may enhance macrophage phagocytic activity and modulate the inflammatory microenvironment, indirectly benefiting bone healing (Parker et al., 2024). Although neurovascular regulation is not a major focus of this review, emerging findings indicate that nerve-mediated vasodilation and growth cues warrant attention.

Collectively, the inflammatory phase is characterized by immune cell–driven initiation of angiogenesis. Accordingly, pro-vascularization strategies often focus on immunomodulation and facilitating early vascular ingress. Immunoregulatory biomaterials—such as platforms that release IL-4 or IL-10 or inhibit NF-κB signaling—can bias macrophages toward an M2-like phenotype, mitigate inflammation, and enhance angiogenesis (Spiller et al., 2015). MSC-derived exosomes also exhibit immunoregulatory and pro-angiogenic functions, positioning them as promising “cell-free therapeutics.” A growing body of work indicates that MSC-derived exosomes can modulate macrophage polarization and promote a shift from M1-like to M2-like states (Si et al., 2025). For example, adipose-derived MSC exosomes enriched in miR-451a can suppress macrophage migration inhibitory factor (MIF) in a rat calvarial defect model, thereby relieving inhibition of anti-inflammatory pathways and promoting M2 polarization (Li et al., 2022).

### 2.2 Angiogenesis in the cartilaginous phase

After inflammation subsides (typically 1–2 weeks post-injury), repair transitions into the cartilaginous phase. In this phase, MSCs within a hypoxic environment differentiate toward chondrocytes and form a fibrocartilaginous bridge that stabilizes the fracture.

The mechanisms of cartilage formation differ across healing modes (Marsell and Einhorn, 2011). In fractures with rigid fixation and small gaps, intramembranous ossification (IMO) predominates: periosteal osteoblasts directly generate bony callus, the cartilage phase is brief or absent, and vascularization and bone formation occur almost concurrently (Benulič et al., 2022). In contrast, unstable fractures and segmental defects typically undergo endochondral ossification (EDO), in which a robust cartilage template forms first; hypertrophic chondrocytes then actively secrete VEGF-A to guide vascular invasion and drive the cartilage-to-bone transition (Marsell and Einhorn, 2011).

Vascular invasion during this stage is governed by tightly orchestrated spatiotemporal control. The timing of vascular entry is critical for healing quality: premature invasion can disrupt proper cartilage template formation and yield an unstable callus, whereas delayed invasion can lead to cartilage necrosis under sustained hypoxia and postpone ossification (Zelzer et al., 2004; Grosso et al., 2023). Early in this stage, proliferating chondrocytes create an avascular cartilage template that restrains early vessel entry. As chondrocytes mature into a hypertrophic state, they upregulate VEGF-A, establishing a concentration gradient at the cartilage–bone interface that attracts vessels from the periphery inward (Zelzer et al., 2004). In parallel, hypertrophic chondrocytes and osteoclast-lineage cells secrete matrix metalloproteinases (MMP-9 and MMP-13), which degrade type II collagen and aggrecan, thereby opening paths for vascular invasion (Stickens et al., 2004). Moreover, osteoclast precursors can release PDGF-BB to induce specialized H-type vessels (CD31^hi^/EMCN^hi^), enabling functional coupling between angiogenesis and osteogenesis (Xie et al., 2014).

Interestingly, in a mouse calvarial injury model—classically representing IMO—bone formation and angiogenesis can be partially “decoupled”: bone tissue formation may precede complete vascular coverage of the defect, while vessels subsequently grow in to fill the region. This temporal sequence differs from the more synchronized vascular–bone progression in long-bone EDO healing (Bixel et al., 2024). These observations suggest that the presence of a cartilaginous phase promotes tighter synchronization between angiogenesis and osteogenesis in long bones, whereas intramembranous repair can permit some degree of bone-first progression. Nonetheless, irrespective of the healing mode, adequate vascular perfusion is ultimately required to ensure bone quality and proper mineralization.

Interventions in the cartilaginous phase primarily aim to support endogenous chondrogenesis and then guide timely vascular invasion. A prominent approach is to engineer biomimetic systems that recapitulate endochondral ossification (Sheehy et al., 2019). Such systems—often porous scaffolds or hydrogels used with cells and instructive cues—can induce MSC chondrogenesis and subsequently promote vascularization and ossification (Sc et al., 2010). For example, loading scaffolds with hypoxia mimetics such as DMOG can stabilize HIF signaling to enhance early cartilage formation (Chen L. et al., 2023), After a cartilage template is established, sequential release of VEGF-A or BMPs can promote timely infiltration of vessels and osteogenic cells, accelerating cartilage-to-bone conversion (Kempen et al., 2009). Notably, no study has yet demonstrated finely tuned temporal coordination of cartilage formation and vascular invasion within a single system solely by regulating scaffold pore size and degradation kinetics. Although delivery modalities and release profiles differ across biomaterial strategies (Benulič et al., 2022), the core principle remains consistent: stabilize the cartilage template first, then direct vascular invasion at an appropriate time.

### 2.3 Angiogenesis in the mineralization phase

The mineralization phase typically occurs during weeks 2–4 post-injury and is characterized by the gradual conversion of the cartilaginous callus into mineralized bone (Baker et al., 2018). A hallmark of this stage is rapid vascular invasion into the cartilaginous callus and the establishment of a dense microvascular network, which supplies oxygen and nutrients required for mineral deposition. As vessels invade, large numbers of osteoblasts—derived from MSCs or via chondrocyte-to-osteoblast transition—migrate together with neovessels into the callus, deposit osteoid, and progressively replace and mineralize the cartilage matrix, ultimately forming woven bone (Yang et al., 2014; Sheen et al., 2025). Angiogenesis and osteogenesis are highly coupled at this stage; the extent and maturity of the vascular network directly determine the pace and quality of callus mineralization (Baker et al., 2018; Kusumbe et al., 2014).

Major cellular players include osteoblasts, osteoclasts, endothelial cells, and bone marrow mesenchymal stem/stromal cells (BMSCs). Osteoblasts localize on the surface of the cartilaginous scaffold and around new vessels, secreting bone matrix and driving mineralization. This process depends on vascular transport of minerals and metabolites. Conversely, osteoblasts can promote angiogenesis by secreting factors such as SLIT3 and BMP-2, thereby reinforcing the osteogenic–vascular unit (Xu et al., 2018). Endothelial cells proliferate and differentiate to build a mature vascular network: tip endothelial cells guide sprout directionality, whereas stalk and branch endothelial cells support vessel elongation and recruit pericytes to stabilize nascent lumens (Lee et al., 2022). Osteoclasts remove cartilage remnants and remodel woven bone while releasing PDGF-BB to promote local angiogenesis (Xie et al., 2014). Meanwhile, BMSCs continuously migrate and differentiate under vascular guidance to sustain osteoblast supply.

VEGF remains a central regulator in this phase, with pronounced dose dependence (Grosso et al., 2023). Appropriate VEGF levels enhance endothelial migration and proliferation and can, via activation of endothelial Notch1 signaling, promote a pro-osteogenic endothelial phenotype. Beyond VEGF/Notch, the Ang/Tie axis also plays key roles: Ang-1 supports vessel maturation and stabilization, whereas Ang-2 participates in remodeling and sprouting (Teichert et al., 2017). Wnt/β-catenin signaling strengthens osteogenic–vascular coupling by regulating osteoblast differentiation and endothelial function (Shen et al., 2021),and PI3K/Akt activation has been shown to increase H-type vessel density in fracture sites (Abdurahman et al., 2022). From a mechanobiological perspective, YAP/TAZ (Hippo pathway) influences osteoblast differentiation and can modulate endothelial HIF-1α expression (Sivaraj et al., 2020).

PDGF-BB is also critical at this stage. Secreted largely by osteoclast-lineage cells, PDGF-BB recruits pericytes through PDGFR-β, stabilizing nascent capillaries and promoting maturation of the vessel–bone structural unit (Xie et al., 2014). Consistently, H-type vessels (CD31^hi^/EMCN^hi^) increase markedly at fracture sites and localize adjacent to osteoblast clusters; their abundance correlates positively with osteogenic activity (Wang et al., 2017).

Interventions during hard callus formation aim to simultaneously enhance angiogenesis and osteogenesis to improve regenerative quality. A widely used strategy is controlled release of pro-angiogenic and pro-osteogenic cues, such as dual-factor delivery systems incorporating VEGF and BMP-2 (Kempen et al., 2009). When released at the appropriate time and dose, VEGF promotes stable neovascularization; however, excessive VEGF can induce aberrant vascular architectures and increase osteoclast activity, thereby reducing net bone formation. Accordingly, many advanced scaffolds adopt a sequential-release design—delivering a lower dose of VEGF early to facilitate vascular invasion, followed by BMP-2 to drive osteoblast differentiation and mineralization—thus avoiding adverse effects associated with sustained, high VEGF exposure (Kempen et al., 2009).

Another important approach is transplantation of pre-vascularized constructs and the use of three-dimensional bioprinting (Nulty et al., 2021). These methods establish microvascular networks *ex vivo* by co-culturing endothelial cells with stem cells, followed by implantation into defects, thereby shortening the time required for functional perfusion. In an Acta Biomaterialia review, De Silva summarized multiple biofabrication strategies for pre-vascularization, including microfluidic generation of perfusable channels, 3D-printed scaffolds with embedded vascular branches, and grafting decellularized vascular networks from animal-derived matrices. Such approaches enable early perfusion during the initial mineralization phase and reduce the risk of ischemic necrosis in the central region of segmental defects (De Silva et al., 2023).

Physical stimulation has also emerged as a complementary modality. In an Advanced Science review, Sun and colleagues highlighted that exogenous electrical stimulation—such as low-intensity pulsed electric fields—or ultrasound can activate Ca^2+^/CaM/NFAT signaling, upregulate VEGF and FGF family factors, promote angiogenesis, and attenuate inflammation (Sun et al., 2025a). More recently, self-powered biodegradable bone-stimulation devices (e.g., implantable nanogenerators) have been designed to convert subtle mechanical deformations of the callus into electrical cues. In animal models, these systems significantly enhance vascularization and increase bone density, and their biodegradability obviates the need for secondary removal surgery (Sun et al., 2025a).

### 2.4 Angiogenesis in the remodeling phase

The remodeling phase is the final stage of fracture healing, typically lasting months to years, and is marked by restoration of bone continuity and gradual recovery of load-bearing function (ElHawary et al., 2021). During this stage, woven bone is progressively replaced by mature lamellar bone, the marrow cavity is reopened, and both mechanical competence and anatomical morphology approach pre-injury states.

Unlike earlier stages that emphasize rapid neovessel expansion, vascular activity in the remodeling phase is dominated by maturation and structural reorganization, often accompanied by a moderate reduction in vessel density. The initially disorganized microvascular network within newly formed woven bone undergoes selective pruning and reconfiguration: some redundant capillaries regress, whereas others enlarge and develop into a Haversian canal–type vascular system that traverses trabecular structures (ElHawary et al., 2021). Using intravital imaging, Bixel and colleagues observed that, in mice, the vascular area fraction decreased from a peak of ∼34%–∼20% during weeks 3–6 post-fracture, indicating that a substantial portion of newly formed vessels are progressively eliminated as the bone integrates. Meanwhile, perfusion patterns gradually normalize, and remaining vessels become aligned with bone structural units to establish a mature intraosseous microcirculation (Bixel et al., 2024).

Bone remodeling is orchestrated by the “basic multicellular unit” (BMU), a coordinated functional ensemble comprising osteoclasts, osteoblasts, and endothelial cells (Bolamperti et al., 2022). Osteoclasts first form a “cutting cone” that resorbs old bone and creates tunnels; neovessels subsequently enter these tunnels, providing oxygen, nutrients, and cellular sources for the following “filling cone” of osteoblasts (Bolamperti et al., 2022). During remodeling, osteoblasts often function as bone-lining or osteoblast-like cells within the BMU and communicate closely with endothelial cells *via* gap junctions (Cx43) and chemokine networks to coordinate vascular remodeling (Kocherova et al., 2019). Osteoblast-derived ANG-1 promotes tight association between Haversian canal endothelium and pericytes (Teichert et al., 2017; Suzuki et al., 2007),while TGF-β and related factors contribute to basement membrane formation and stabilize remodeled vessels (Kemp et al., 2022).

Pericytes play a pivotal role in vascular maturation. PDGFR-β^+^ pericytes derived from bone marrow stroma or vessel walls progressively ensheath nascent vessels and can transition toward smooth muscle–like phenotypes, reinforcing vascular stability (Vontell et al., 2006). When HIF-1α/PDGF-BB/PDGFR-β signaling weakens, pericytes may detach from the vessel wall, leading to vascular instability and injury (Vontell et al., 2006; Gaengel et al., 2009).

Vascular remodeling is also intertwined with restoration of hematopoiesis. Hematopoietic stem cells (HSCs) return to perivascular niches; endothelial cells sustain hematopoietic function by secreting stem cell factor (SCF), whereas hematopoietic cells secrete cytokines such as IL-6 that influence local bone metabolism (Ding et al., 2012). Although this vascular–hematopoietic crosstalk is not the principal driver of bone remodeling, it underscores the broader importance of vascular reconstruction for marrow functional recovery.

Overall, the remodeling phase represents a transition from rapid, coarse vascular expansion to refined, structured reorganization of the bone–vascular network, with the overarching goal of optimizing bone quality and functional architecture. From a biomaterials perspective, scaffolds or graft substitutes used earlier should ideally degrade progressively during remodeling to avoid foreign-body interference with structural adaptation (Dec et al., 2022). Importantly, this consideration is directly relevant to exosome-enabled pro-angiogenic therapy, because biodegradable hydrogels and porous scaffolds are commonly used as local EV depots to prolong retention and enable sustained release (see Section 5.1); therefore, scaffold degradation kinetics shape the temporal profile of exosome-derived angiogenic cues during vessel maturation and pruning. Optimal materials provide mechanical support and promote early vascular invasion while fully resorbing in the remodeling phase without residuals that impair bone architecture (Dec et al., 2022). Materials with tunable degradation—such as β-tricalcium phosphate (β-TCP) and bioactive glass (Bioglass)—have been developed, and their degradation products may further stimulate remodeling. In addition, absorbable scaffolds incorporating functional elements (e.g., piezoelectric fibers or magneto-responsive particles) have been explored; these can be activated by external physical fields during remodeling to deliver subtle stimuli that increase bone density (Meng et al., 2013).

### 2.5 Stage-tailored design of EV-based pro-angiogenic therapy

Because angiogenesis is dynamically coupled to inflammation, chondrogenesis, mineralization, and remodeling, EV-based interventions should be synchronized with phase-specific vascular bottlenecks rather than applied as a uniform “pro-angiogenic” stimulus across the entire repair course (Marsell and Einhorn, 2011). A pragmatic design logic is to define for each phase: (i) the dominant vascular task (sprouting initiation, cartilage invasion, network maturation, or pruning), (ii) the principal receiver cell states (e.g., macrophages, endothelial tip/stalk cells, chondrocyte-lineage cells, osteoprogenitors, and pericytes), and (iii) the EV sender source and cargo modules most likely to relieve the bottleneck, while matching the delivery format to the desired exposure window (bolus versus depot/sustained release).

Inflammatory phase (hours–days): The primary objective is to temper excessive inflammation while permitting early endothelial activation and sprouting. Macrophage phenotypes are key determinants of vascularization (Spiller et al., 2014); therefore, early EV therapy should prioritize immune reprogramming that establishes a permissive pro-angiogenic milieu. For example, adipose-derived stem cell exosomes have been shown to promote bone healing by shifting macrophage polarization through a miR-451a/MIF axis (Li et al., 2022). In this short time window, localized bolus dosing or short-lived depots may be preferred to avoid prolonged immunosuppression and to better align with the transient nature of inflammatory signaling.

Cartilaginous (soft callus) phase: In endochondral repair, the avascular cartilage template must first be stabilized, followed by timely vascular invasion. VEGF-A signaling is crucial for chondrocyte survival and cartilage vascularization (Zelzer et al., 2004), yet premature or excessive angiogenic stimulation can disturb chondrogenesis and the cartilage-to-bone transition. Thus, stage-aware strategies that delay or sequence angiogenic cues—conceptually analogous to sequential VEGF/BMP-2 delivery—are more consistent with endochondral biology (Kempen et al., 2009). Practically, this can be implemented by selecting EVs enriched in hypoxia-regulated pro-angiogenic miRNAs and delivering them preferentially in late soft callus; hypoxia-conditioned stem cell exosomes, for instance, can enhance endothelial angiogenic responses *via* transfer of let-7f-5p and miR-210–3p (Liu et al., 2022).

Mineralization (hard callus) phase: Rapid expansion and maturation of a functional microvascular network becomes rate-limiting for mineral deposition and coupled osteogenesis. Mechanistically, the specialized CD31^hi^Emcn^hi^ (type-H) endothelium provides an angiogenic niche that couples angiogenesis to osteogenesis (Kusumbe et al., 2014), and PDGF-BB secreted by preosteoclasts induces angiogenesis during this coupling process (Xie et al., 2014). Accordingly, EV sources and cargos at this stage can be tuned toward robust endothelial sprouting/maturation together with osteogenic coupling. Hypoxia-preconditioned MSC-sEVs enriched in miR-210-3p have been shown to enhance vascularized bone regeneration (Zhuang et al., 2022), and adipose-derived stem cell exosomal miR-21-5p can stimulate angiogenesis in endothelial progenitor cells to promote bone repair (Cao et al., 2024). Because this phase spans weeks, sustained delivery formats that maintain local exposure are advantageous; thermosensitive hydrogel-encapsulated MSC-sEVs, for example, can prolong local release and improve angiogenesis and bone regeneration *via* exosomal miR-21 signaling (Wu D. et al., 2022).

Remodeling phase: Vascular demands shift from maximal expansion to stabilization, pruning, and integration with BMU-driven remodeling. Direct evidence for stage-timed EV dosing in this late window remains limited; nevertheless, EV strategies could be designed to favor vessel stabilization (e.g., pericyte recruitment/coverage and anti-leakage signaling) and balanced osteoclast–osteoblast coupling rather than continued sprouting, highlighting a key priority for future longitudinal, stage-resolved EV studies.

## 3 Characteristics and therapeutic potential of stem cell-derived extracellular vesicles

### 3.1 Characteristics of stem cell–derived extracellular vesicles

**FIGURE 2:**
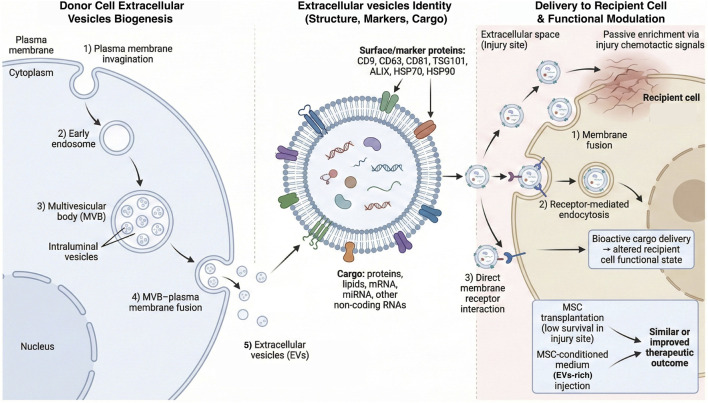
Extracellular vesicle (EV) biogenesis, molecular identity, and cell-free mechanism in bone repair. Left: EV the biogenesis via endosomal pathway. Plasma-membrane invagination forms early endosomes that mature into multivesicular bodies (MVBs) containing intraluminal vesicles (ILVs); fusion of MVBs with the plasma membrane releases ILVs as small EVs/exosomes. Middle: representative EV architecture with a lipid bilayer, commonly used identity markers (e.g., CD9, CD63, CD81, TSG101, ALIX, HSP70 and HSP90) and major cargo classes (proteins, lipids, mRNAs, miRNAs and other non-coding RNAs). Right: delivery to recipient cells at the injury site. EVs can accumulate in injured tissues and enter recipient cells through membrane fusion, receptor-mediated endocytosis, or ligand–receptor interactions, enabling cytosolic delivery of bioactive cargo that reprograms recipient cell states. The inset contrasts low survival of transplanted MSCs at injury sites with EV-rich MSC-conditioned medium as a cell-free alternative. Created by the authors based on Raposo and Stoorvogel (2013), van Niel et al. (2018), Théry et al. (2018), and Mathieu et al. (2019). Abbreviations: ALIX, ALG-2-interacting protein X; EV, extracellular vesicle; HSP, heat shock protein; ILV, intraluminal vesicle; miRNA, microRNA; mRNA, messenger RNA; MSC, mesenchymal stromal/stem cell; MVB, multivesicular body; TSG101, tumor susceptibility gene 101.

Extracellular vesicles (EVs) are lipid-bilayer particles naturally released by cells and capable of transferring bioactive cargos to recipient cells. In the context of most regenerative studies, the best-characterized fraction is small extracellular vesicles (sEVs)—typically in the ∼30–150/200 nm range—often enriched through widely used isolation workflows and historically referred to as “exosomes” in many reports (Kalluri and LeBleu, 2020). sEVs can arise *via* the endocytic pathway: early endosomes mature into multivesicular bodies (MVBs), and fusion of MVBs with the plasma membrane releases intraluminal vesicles into the extracellular space as sEVs (Van Niel et al., 2018). Commonly used markers include tetraspanins (CD9, CD63, CD81) and endosomal proteins (TSG101, ALIX), together with heat-shock proteins (e.g., HSP70 and HSP90) (Théry et al., 2018). Their cargos—proteins, lipids, mRNAs, microRNAs (miRNAs), and other non-coding RNAs—enable EVs/sEVs to function as intercellular messengers *via* membrane fusion, endocytosis, or ligand–receptor interactions, ultimately reprogramming gene expression and cellular states in recipient cells (Gurung et al., 2021) (Figure 2).

Stem cell–derived extracellular vesicles have attracted substantial attention because they can inherit—and transmit—key regenerative properties of their parent cells. Among these, MSCs–derived extracellular vesicles are particularly prominent: they carry regeneration-associated signals characteristic of MSCs and can convey reparative capacity to recipient cells (Kou et al., 2022). Although transplanted MSCs typically exhibit poor survival and limited long-term persistence or differentiation within injured tissues, extensive evidence indicates that MSCs can nonetheless markedly improve tissue repair—largely through biological actions mediated by paracrine factors, including extracellular vesicles (Kou et al., 2022). Consistently, both *in vitro* and *in vivo* studies have shown that administration of MSC-conditioned medium can achieve therapeutic efficacy comparable to—or even exceeding—that of MSC transplantation, underscoring extracellular vesicles as major effectors of stem cell–based therapies (Gnecchi et al., 2006).

Here, we use MSC transplantation as a clinically relevant cell-based benchmark because MSC-derived EVs are widely considered major effectors of MSC paracrine repair. Compared with direct stem‐cell transplantation, cell-free therapies based on stem cell–derived extracellular vesicles (EVs) offer several clear advantages.

1. Cell-free composition enables greater product standardization. EVs are membrane-enclosed nanoparticles and do not require the recovery, viability maintenance, or functional preservation of living cells. As a result, standardization is more feasible across formulation/dosage form development, transportation, and clinical administration, facilitating “off-the-shelf” deployment.
2. No capacity for proliferation or differentiation, reducing long-term safety concerns linked to live-cell persistence and ectopic differentiation. EVs are anucleate and cannot replicate. Unlike transplanted cells, they do not engraft long term or undergo differentiation *in vivo*, thereby mechanistically lowering the risk of ectopic differentiation and other delayed safety liabilities associated with live-cell therapies.
3. Nanoscale size favors local diffusion and cellular uptake. The nanometer-scale dimensions of EVs support diffusion within local microenvironments and efficient endocytosis. In localized delivery settings (e.g., defect-site administration or sustained release from scaffolds/hydrogels), this can increase tissue retention and prolong effective exposure.
4. Engineering enables active targeting and functional enhancement. EVs can be engineered through donor-cell manipulation (e.g., modulating specific miRNA/protein loading or expressing membrane protein–peptide fusions) and through post-isolation chemical/physical modifications to augment cargo and confer active targeting. A classic proof-of-concept study expressed a Lamp2b–RVG fusion protein in dendritic cells and loaded siRNA into EVs; following intravenous injection, the EVs delivered cargo to mouse brain cells and achieved gene silencing, supporting the feasibility of ligand-mediated active targeting (Alvarez-Erviti et al., 2011).

In addition to cell transplantation, EV-based pro-angiogenic therapies should be interpreted alongside other pro-angiogenic biomaterials and nanoscale carriers used in vascularized bone repair, including growth-factor–releasing scaffolds/hydrogels (Kempen et al., 2009; Lu et al., 2024), liposomes or polymeric/inorganic nanoparticles for protein/miRNA delivery, and EV-inspired nanocarriers such as exosome-membrane–coated nanoparticles (Poinsot et al., 2024; Lopes et al., 2023). Compared with many synthetic nanoparticles that typically deliver single defined cargos with high loading capacity and tunable release, EVs naturally package combinatorial miRNAs, proteins, and lipids and present native membrane ligands that facilitate cellular uptake and may provide multi-pathway instruction (Witwer and Wolfram, 2021). Conversely, synthetic nanocarriers often offer superior compositional control and manufacturing scalability, whereas EVs face challenges of biological heterogeneity and more complex quality control. This added comparison better positions EVs within the broader landscape of pro-angiogenic biomaterial strategies for bone regeneration (Kang et al., 2021).

### 3.2 Bone-repair potential of stem cell–derived extracellular vesicles

**FIGURE 3:**
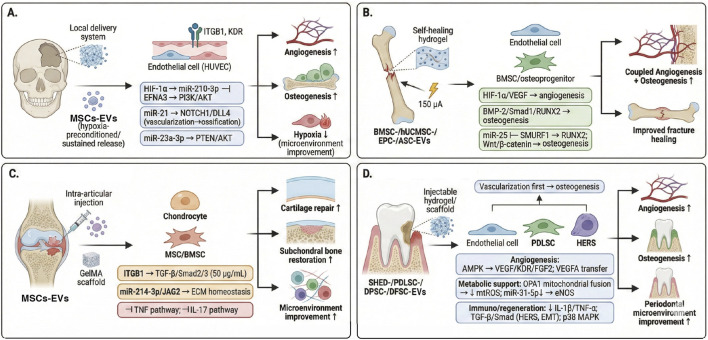
Stem cell-derived EVs for bone repair: model-specific delivery settings and dominant mechanisms. **(A)** Cranial critical-sized defect: local delivery of MSC-EVs (often hypoxia-preconditioned or formulated for sustained release) targets endothelial cells (e.g., HUVECs) and osteoprogenitors to enhance angiogenesis and osteogenesis and improve hypoxic microenvironments; representative axes include HIF-1α–miR-210-3p–EFNA3/PI3K–AKT and miR-21–NOTCH1/DLL4 signaling. **(B)** Long-bone fracture/segmental defect: EVs derived from BMSC, hUCMSC, EPC or ASC are delivered using self-healing hydrogels (with or without microcurrent stimulation, 150 μA) to promote coupled angiogenesis (HIF-1α/VEGF) and osteogenesis (BMP-2/Smad1/RUNX2; Wnt/β-catenin). **(C)** Osteochondral defect: intra-articular EV delivery, optionally supported by GelMA scaffolds, acts on chondrocytes and stromal cells to support cartilage repair and subchondral bone restoration, in part via ITGB1–TGF-β/Smad2/3 and ECM-homeostatic cargo (e.g., miR-214-3p/JAG2) with suppression of inflammatory pathways (e.g., TNF and IL-17 signaling). **(D)** Alveolar/periodontal defect: EV-laden injectable hydrogels/scaffolds (e.g., from SHED, PDLSC, DPSC or DFSC) prioritize early vascularization followed by osteogenesis; reported mechanisms include AMPK–VEGF signaling, mitochondrial support (OPA1/mtROS) and immunomodulation (reduced IL-1β/TNF-α), including effects on HERS-associated epithelial–mesenchymal programs during periodontal repair. Created by the authors by integrating representative preclinical studies (Liu et al., 2020; Wu et al., 2019; Wu et al., 2022; Zhao et al., 2022; Zhang et al., 2022). Abbreviations: AMPK, AMP-activated protein kinase; ASC, adipose-derived stromal/stem cell; BMSC, bone marrow mesenchymal stromal/stem cell; BMP-2, bone morphogenetic protein 2; DFSC, dental follicle stem cell; DPSC, dental pulp stem cell; EC, endothelial cell; ECM, extracellular matrix; eNOS, endothelial nitric oxide synthase; EMT, epithelial–mesenchymal transition; EPC, endothelial progenitor cell; EFNA3, ephrin-A3; FGF2, fibroblast growth factor 2; GelMA, gelatin methacrylate; HERS, Hertwig’s epithelial root sheath; HIF-1α, hypoxia-inducible factor-1α; hUCMSC, human umbilical cord mesenchymal stromal/stem cell; HUVEC, human umbilical vein endothelial cell; IL, interleukin; ITGB1, integrin β1; JAG2, Jagged 2; KDR, kinase insert domain receptor (VEGFR2); MAPK, mitogen-activated protein kinase; miR, microRNA; mtROS, mitochondrial reactive oxygen species; NOTCH1, notch receptor 1; OPA1, optic atrophy 1; PDLSC, periodontal ligament stem cell; PI3K, phosphoinositide 3-kinase; PTEN, phosphatase and tensin homolog; RUNX2, runt-related transcription factor 2; SHED, stem cells from human exfoliated deciduous teeth; Smad, SMAD family proteins; SPRY2, sprouty homolog 2; TGF-β, transforming growth factor-β; TNF, tumor necrosis factor; VEGF, vascular endothelial growth factor; VEGFR2, VEGF receptor 2; Wnt, Wingless/Int.

Building on these advantages, stem cell–derived EVs have shown therapeutic effects comparable to, and in some contexts exceeding, those of cell-based approaches across multiple tissue-repair models. In bone regeneration, the efficacy of mesenchymal stromal/stem cell–derived small EVs (MSC-sEVs) has also been widely documented in diverse bone and skeletal defect models, although outcomes can vary modestly depending on the tissue context (Figure 3).

#### 3.2.1 Critical size calvarial/parietal defects (intramembranous ossification–dominant)

A key bottleneck in repairing large cranial defects is insufficient vascular regeneration, which creates an ischemic and hypoxic microenvironment. This milieu not only constrains new bone formation but also compromises graft survival and functional reconstruction. Accordingly, achieving robust angiogenesis early during cranial repair has become a central strategy to improve regenerative quality.

Hypoxic preconditioning is an effective approach to potentiate the angiogenic capacity of MSC-derived sEVs. Zhuang et al. generated sEVs from MSCs cultured under hypoxia and found that these sEVs markedly promoted proliferation, migration, and tube formation of human umbilical vein endothelial cells (HUVECs) (Zhuang et al., 2022). *In vivo*, they further improved neovascularization and osteogenesis in cranial defect sites. Mechanistically, hypoxia induced HIF-1α–dependent upregulation of miR-210–3p, which promoted angiogenesis by suppressing *EFNA3* and activating the PI3K/AKT pathway.

Second, integrating exosomes with sustained-release scaffolds and hydrogels to extend local exposure and optimize release kinetics represents another major direction. Zhang et al. encapsulated umbilical cord MSC–derived exosomes in a hyaluronic acid hydrogel and combined this system with a nano-hydroxyapatite/polycaprolactone (nHA/PCL) scaffold, enabling controlled local release. In a rat cranial defect model, this strategy enhanced both angiogenesis and bone regeneration; mechanistic analyses implicated exosomal miR-21 and activation of NOTCH1/DLL4 signaling, underscoring a sequential paradigm of “vascularization first, ossification next” (Zhang Y. et al., 2021). Similarly, Zhang et al. combined induced pluripotent stem cell–derived mesenchymal stromal/stem cell (iPSC-MSC) exosomes with a β-tricalcium phosphate (β-TCP) scaffold, which upregulated PI3K/AKT signaling and osteogenic gene programs in recipient bone marrow mesenchymal stromal/stem cells (BMSCs) (Zhang J. et al., 2016). In ovariectomized (OVX) rats with cranial defects, Qi et al. reported a dose-dependent increase in micro-computed tomography (micro-CT)–quantified bone formation and Microfil perfusion–assessed angiogenesis following iPSC-MSC exosome administration, with further enhancement when the exosomes were incorporated into a β-TCP scaffold (Qi et al., 2016).

Along the same lines, Hu et al. incorporated human umbilical cord MSC–derived sEVs into a bioactive glass–GelMA hydrogel (BG gel–sEVs). This system leveraged pro-angiogenic miRNAs (e.g., miR-23a-3p) and sustained release to activate the PTEN/AKT axis, thereby strengthening early angiogenesis and indirectly promoting osteogenesis—highlighting the advantage of miRNA-oriented targeted delivery within controlled-release matrices (Hu et al., 2022).

Beyond biomaterial strategies, genetic modification of donor cells to enhance exosomal function has also yielded substantial benefits. Ying et al. combined β-TCP scaffolds with exosomes from BMSCs expressing a triple-mutant HIF-1α, achieving significantly greater neovascularization and new bone formation than with conventional BMSC exosomes. Mechanistically, stabilized HIF-1α signaling not only promoted angiogenesis directly but also recruited more endothelial progenitor cells (EPCs) to the defect site, thereby improving repair efficiency (Ying et al., 2020).

Finally, driving donor cells into specific differentiation states to generate EVs enriched with regeneration-relevant proteins or miRNAs may improve the consistency and reliability of bone regeneration. Al Sharabi et al. compared EVs from uninduced MSCs *versus* early osteogenically induced MSCs and found that the latter were enriched in bone-associated proteins and more strongly promoted MSC osteogenic differentiation. In cranial defect models, these EVs also produced more stable pro-regenerative outcomes, suggesting that donor-cell state can encode intrinsic “regenerative instructions” within EV cargo (Al-Sharabi et al., 2024).

#### 3.2.2 Long-bone fracture and segmental defect models (endochondral ossification–dominant)

Effective repair of long-bone fractures and critical-size segmental defects depends heavily on early and sustained microvascular reconstruction. Accordingly, several prevascularization strategies have been developed to improve outcomes in segmental defects. Nulty et al. used 3D bioprinting to pre-build a microvascular network composed of HUVECs and hBMSCs within a printable hydrogel matrix; after implantation, this construct markedly accelerated vascular ingrowth and bone regeneration within the defect site (Nulty et al., 2021). Xu et al. proposed an *in vivo* prevascularization approach in which a β-tricalcium phosphate (β-TCP) scaffold was first prevascularized within the vastus lateralis muscle before being transferred to the bone defect, enabling rapid establishment of a stable vascular network and improved repair (Xu et al., 2022).

In contrast to these prevascularization methods, EV–based strategies are geared toward unlocking endogenous angiogenic capacity, with favorable biocompatibility and translational potential. Exosomes/small EVs from multiple stem/progenitor sources (e.g., BMSC-Exos, hUCMSC-Exos, ASC-Exos, EPC-Exos) can enhance repair by directly augmenting endothelial function and angiogenesis, reshaping the metabolic milieu of the fracture microenvironment, and activating coupled osteogenic–angiogenic signaling axes, ultimately improving bone mass as well as the mechanical and histological quality of healing.

In a rat femoral stabilized fracture model, Zhang et al. reported that hUCMSC-Exos significantly enhanced endothelial tubulogenesis and *in vivo* neovascularization by upregulating the HIF-1α/VEGF pathway, thereby increasing vascular density and improving tissue repair quality at the fracture site (Zhang Y et al., 2019). Similarly, Jiang et al. (2020) showed that miR-25 enriched in BMSC-Exos suppressed *SMURF1*-mediated ubiquitination and degradation of *RUNX2*, resulting in a pronounced acceleration of fracture healing in mice (Jiang et al., 2020). In a tibial distraction osteogenesis model, Jia et al. demonstrated that EPC-Exos markedly promoted HUVEC proliferation, migration, and tube formation and accelerated new bone formation *in vivo*, in a miR-126–dependent manner (Jia et al., 2019). Moreover, in a rat femoral nonunion model, BMMSC-derived exosomes activated both BMP2/Smad1/RUNX2 and HIF-1α/VEGF signaling, coordinately promoting osteogenesis and angiogenesis (Zhang L. et al., 2020).

Beyond direct endothelial effects, stem cell exosomes can also reprogram the local metabolic microenvironment, thereby reinforcing bone–vascular coupling during repair. Using metabolomics, Li et al. found that BMSC-Exos promoted synchronous angiogenesis and bone regeneration by modulating host systemic metabolic programs, including HIF-1–related and lipid metabolism pathways. This metabolic remodeling may not only secure adequate energetic support for regeneration but could also potentiate repair through epigenetic mechanisms (Li C. et al., 2025). Consistently, Ying et al. further showed that ASC-Exos enhanced the coupling of fracture healing and angiogenesis via activation of the Wnt/β-catenin pathway; causality was supported by pathway blockade using the exosome inhibitor GW4869 (Zhang D. et al., 2023).

Delivery modality and optimization are also pivotal. Wang et al. developed a self-healing hydrogel loaded with hUCMSC-derived exosomes to enable sustained and stable release within the defect region, significantly improving local vascularization and bone regeneration (Wang L. et al., 2020). Chen et al. introduced an electrical stimulation preconditioning strategy and optimized parameters (150 μA) to enrich BMSC-exosomal cargo with pro-osteogenic and pro-angiogenic factors, resulting in markedly improved repair in a femoral defect model (Chen et al., 2025). Collectively, these studies indicate that the efficacy of exosome-based therapy hinges not only on intrinsic bioactivity, but also on delivery vehicles, release kinetics, and precisely programmed cargo composition.

#### 3.2.3 Osteochondral defect models

Osteochondral defects remain a major clinical challenge because successful repair requires coordinated regeneration of articular cartilage and the highly vascularized subchondral bone. Although cartilage is avascular, timely revascularization and vascular remodeling within the subchondral compartment are indispensable for rebuilding the osteochondral unit and can indirectly support cartilage homeostasis via nutrient delivery and angiocrine signaling (Wei and Dai, 2021). In this context, stem cell–derived extracellular vesicles (EVs) have emerged as a promising cell-free therapeutic modality for osteochondral restoration, providing a relevant setting to interrogate EV-driven angiogenesis–osteogenesis coupling alongside cartilage repair.

In a rat knee osteochondral defect model, weekly intra-articular administration of human embryonic MSC-derived exosomes for 12 weeks led to the formation of hyaline-like cartilage and restoration of near-normal subchondral bone architecture within the defect (Zhang S. et al., 2016). Efficacy has also been demonstrated in clinically relevant large-animal models. In a mini-pig osteochondral defect study, three weekly intra-articular injections of MSC exosomes combined with hyaluronic acid produced superior MRI-detected cartilage repair as early as 2 weeks compared with controls; at 4 months, the exosome-treated group outperformed hyaluronic acid alone in gross appearance, histological scores, and biomechanical properties (Zhang et al., 2022). Chen et al. further reported that human Wharton’s jelly MSC–derived EVs (hWJMSC-EVs) significantly improved cartilage regeneration in a rabbit osteochondral defect model via an ITGB1-mediated TGF-β/Smad2/3 axis, and identified an optimal dose of 50 μg/mL (Chen Z. et al., 2024).

Mechanistically, stem cell exosomes can facilitate osteochondral repair by improving the local tissue milieu and enhancing cellular functions. Lou et al. integrated a 3D-printed GelMA scaffold with infrapatellar fat pad–derived synovial stem cell exosomes (SSC-Exos), achieving robust, synchronous repair across the cartilage–subchondral bone interface. This effect was linked to the miR-214-3p/*JAG2* axis, influencing proliferation, migration, extracellular matrix (ECM) homeostasis, and immunomodulation (Lou et al., 2025). In another study, Chen et al. (2024) showed that cartilage stem/progenitor cell–derived exosomes (CSPC-Exos) mitigated osteoarthritis progression by suppressing inflammatory pathways (TNF and IL-17 signaling) while enhancing metabolic repair programs (Chen J. et al., 2024).

Delivery format is a key determinant of therapeutic performance. Liu et al. used a photocurable adhesive hydrogel to localize iPSC-MSC exosomes, significantly promoting neocartilage formation in a rabbit cartilage defect model (Liu et al., 2017). Jiang et al. reported that combining hWJMSC exosomes with an acellular cartilage ECM scaffold (ACECM) improved repair quality through immunomodulation and miRNA-driven ECM synthesis (Jiang et al., 2021). Chen et al. (2019) developed a radially oriented ECM/GelMA scaffold system that enabled sustained exosome release and restoration of mitochondrial homeostasis, thereby improving cartilage repair (Chen et al., 2019). Yan et al. (2023) further suggested that exosomes produced under 3D culture conditions (3D-Exos) outperform conventional 2D-derived exosomes by enhancing MSC recruitment, chondrogenic induction, and immunomodulatory capacity (Yan et al., 2023).

#### 3.2.4 Alveolar bone defect models

Alveolar bone is a tooth-supporting and tooth-dependent bone integrated with the periodontal ligament/cementum complex, and it shows distinct developmental/anatomical and functional features compared with other skeletal sites (Liang et al., 2020). The periodontal ligament provides vascular supply and nutrients to the alveolar bone, making revascularization a foundational requirement for functional periodontal/alveolar repair (Jiang et al., 2016). Healing of extraction sockets involves early vascular sprouting, moreover, in non-contained (e.g., supra-alveolar) periodontal defects, limited vascularity is recognized as a key obstacle to regeneration (Wang et al., 2023; Shakya et al., 2024).

Stem cell exosomes can stimulate endothelial proliferation, migration, and tubulogenesis, increasing microvessel density and thereby improving alveolar defect repair. For example, exosomes derived from stem cells of human exfoliated deciduous teeth (SHED) were shown to upregulate key angiogenic mediators in endothelial cells, including *VEGF, KDR* (*VEGFR2*), and *FGF2*, and significantly enhance tube formation; mechanistic analyses implicated the AMPK pathway (Wu et al., 2019). Another study reported that exosomes from SHED aggregates were enriched in miR-222, which enhanced angiogenesis and matrix remodeling activity in periodontal ligament stem cells (PDLSCs) and increased new bone volume and vascular density *in vivo* (Zhou et al., 2025). PDLSC-derived exosomes can also promote endothelial angiogenesis through transfer of VEGF-A protein, with particularly pronounced effects under inflammatory conditions (Zhang Z. et al., 2020).

Exosomes may further support angiogenesis by maintaining endothelial energy metabolism homeostasis. Lu et al. showed that exosomes restored endothelial mitochondrial function and metabolic stability by promoting *OPA1*-mediated mitochondrial fusion and reducing mtROS, thereby improving angiogenesis and bone regeneration in a diabetic alveolar bone defect model (Lu et al., 2025). In addition, PDLSC exosomes were reported to downregulate miR-31-5p, relieving suppression of eNOS and indirectly enhancing local angiogenesis and osteogenesis (Lu et al., 2023).

Immunomodulation represents another key axis. DPSC-derived exosomes reduced inflammatory cytokines (e.g., IL-1β and TNF-α), attenuated osteoclastic activity, improved the periodontal microenvironment, and promoted bone repair (Qiao et al., 2023). Moreover, DPSC exosomes promoted HERS cell proliferation and epithelial–mesenchymal transition (EMT) via TGF-β/Smad signaling, supporting alveolar bone regeneration (He et al., 2024). Exosomes/sEVs from DFSCs activated p38 MAPK to promote PDLSC osteogenesis and migration, facilitating periodontal structural reconstruction (Ma et al., 2022).

Rational biomaterials and tissue-engineering approaches can further amplify therapeutic benefit by enabling local enrichment, spatial confinement, and temporally controlled release of exosomes. Injectable hydrogels have become dominant platforms: a thermosensitive injectable gel enabled early exosome release and improved angiogenesis and bone regeneration in diabetic alveolar defects (Lu et al., 2025); an Alg-Gel composite hydrogel enhanced the pro-osteogenic and pro-angiogenic effects of PDLSC exosomes (Zhao Y. et al., 2022); and a PF127 hydrogel loaded with *CTNNB1*-modified exosomes increased new bone formation while exerting anti-inflammatory effects (He et al., 2023).

Beyond hydrogels, 3D printing and composite scaffolds offer structured carriers for dose control and spatial patterning, improving vascular invasion and osteogenesis (Sun et al., 2023); A coaxial SIS/HA composite scaffold enabling rapid antimicrobial peptide release together with slow, sustained EV release showed excellent reparative efficacy under complex infectious/inflammatory conditions (Ma et al., 2025). β-TCP combined with a barrier membrane and EV loading further improved angiogenesis and collagen remodeling (Wu et al., 2019).

Finally, to address stability and scalable manufacturing, approaches such as ultrasound stimulation and metal–polyphenol network (MPN) technologies have been introduced to facilitate large-scale EV production and protect vesicles under inflammatory pathophysiological conditions (Zhang T. et al., 2023; Zhou et al., 2026).

## 4 Cargo profiles and functional pathways of stem cell–derived EVs in angiogenesis-driven bone regeneration

**FIGURE 4:**
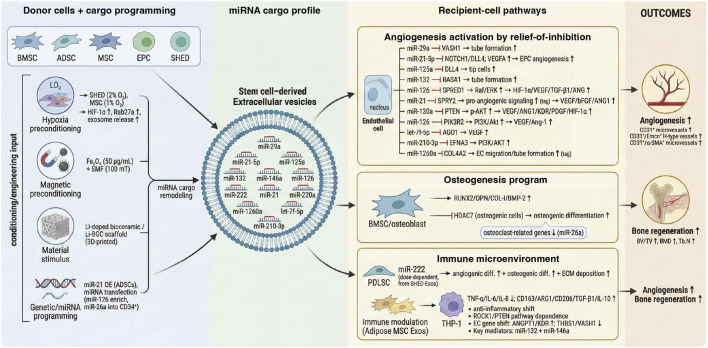
Cargo programming and recipient-cell pathways of stem cell–derived EVs in angiogenesis-driven bone regeneration. Left: donor-cell sources (e.g., BMSC, ADSC, MSC, EPC, SHED) and conditioning/engineering inputs (hypoxia, magnetic nanoparticle + static magnetic field stimulation, bioactive ion-releasing scaffolds, or genetic/miRNA programming) reshape EV cargo. Middle: representative EV miRNA cargo (examples shown) illustrates combinatorial packaging rather than single-agent delivery. Right: selected recipient-cell programs mapped to endothelial angiogenesis, osteogenesis, and immune microenvironment modulation through reported miRNA–target interactions that converge on Notch/DLL4, PI3K–AKT, Ras–ERK/HIF-1α/VEGF and Ang/Tie signaling in endothelial cells, and RUNX2/OPN/COL-I/BMP-2 programs in osteogenic cells, while promoting a pro-repair macrophage phenotype. Outcomes include increased CD31hi/EMCNhi type-H vasculature and improved bone microarchitecture (e.g., BV/TV, BMD and Tb.N). Created by the authors based on representative mechanistic studies of EV “preconditioning” and miRNA-mediated coupling of angiogenesis–osteogenesis (Liu et al., 2019; Liu et al., 2020; Wu et al., 2021; Gao et al., 2022). Abbreviations: ADSC, adipose-derived stromal/stem cell; AGO1, Argonaute 1; Ang, angiopoietin; BMD, bone mineral density; BMP-2, bone morphogenetic protein 2; BMSC, bone marrow mesenchymal stromal/stem cell; BV/TV, bone volume/total volume; CD31hi/EMCNhi, type-H vessels; COL-I, type I collagen; COL4A2, collagen type IV alpha 2 chain; DLL4, Delta-like ligand 4; EC, endothelial cell; EFNA3, ephrin-A3; EPC, endothelial progenitor cell; ERK, extracellular signal-regulated kinase; Fe3O4, magnetite nanoparticles; HIF-1α, hypoxia-inducible factor-1α; HDAC7, histone deacetylase 7; KDR, VEGFR2; miR, microRNA; MSC, mesenchymal stromal/stem cell; OPN, osteopontin; PDGFR, platelet-derived growth factor receptor; PI3K, phosphoinositide 3-kinase; PTEN, phosphatase and tensin homolog; RUNX2, runt-related transcription factor 2; SHED, stem cells from human exfoliated deciduous teeth; SMF, static magnetic field; Tb.N, trabecular number; TGF-β, transforming growth factor-β; THBS1, thrombospondin-1; VEGF(A), vascular endothelial growth factor (A); VASH1, vasohibin-1.

Stem cell–derived EVs carry a multimodal cargo repertoire—including miRNAs, other regulatory RNAs, proteins, lipids, and metabolites—that can coordinately reprogram endothelial activation, immune tone, and osteogenic differentiation. In the context of angiogenesis-driven bone regeneration, mechanistic causality has been most consistently established for miRNAs, because individual miRNAs can be experimentally enriched or depleted in EVs and linked to defined targets in vascular responder cells together with *in vivo* vascular and bone outcomes. Therefore, this section summarizes representative, mechanistically validated pro-angiogenic miRNAs (Table 1; Figure 4), selected based on (i) evidence of EV-mediated transfer into recipient cells, (ii) gain- or loss-of-function validation of targets or pathways, and (iii) angiogenic/osteogenic phenotypes in relevant models.

**TABLE 1:** Key cargo profiles and functional pathways of stem/stromal cell-derived EVs promoting angiogenesis and bone regeneration.

Cargo	EV source (cell/Strategy)	Target(s)	Pathway/Mechanism	*In vivo* model	Dose/Regimen (route; frequency)	Main outcomes (angiogenesis/Osteogenesis)
miR-29a (Lu et al., 2020)	BMSC-derived exosomes (miR-29a-enriched)	*VASH1*↓	Relief of anti-angiogenic signaling; angio-osteogenic coupling	Mouse bone phenotype model (micro-CT; vessel density)	Tail vein: 100 μg in 100 μL PBS, twice/week for 2 months	↑Tube formation; ↑BV/TV, BMD, Tb.N; ↑neovessel density
miR-21-5p (Cao et al., 2024)	ADSC-derived exosomes	*NOTCH1/DLL4*↓; *VEGFA*↑	NOTCH1/DLL4/VEGFA signaling	EPC angiogenesis *in vitro*; cranial defect repair with HA-gel delivery	Exosomes in HA gel: 5 or 20 μg/mL (volume NR)	↑Angio-osteogenesis: micro-CT (↑BV/TV, BMD, etc.); IHC (↑CD31/VEGFA and osteogenic markers)
miR-125a (Liang et al., 2016)	Human adipose MSC-derived exosomes	*DLL4*↓	Relieves Notch–DLL4 restraint; increases tip-cell specification	Tube formation *in vitro*; Matrigel plug angiogenesis (nude mice; HUVECs pretreated)	HUVEC pretreatment: exosomes 100 μg/mL; Matrigel plug: 3 × 10^6^ cells/200 μL + Matrigel 200 μL	Tip cells ∼11%→22%; ↑tube formation/vessel structures (CD31 staining)
miR-126 (Jia et al., 2019)	EPC-derived exosomes	*SPRED1*↓	Raf/ERK activation; ↑HIF-1α/VEGF/TGF-β1/ANG, etc.	Distraction osteogenesis model	Local injection: 1 × 10^11^ exosomes in 100 μL PBS (PBS control)	↑Vessel volume fraction and osteogenesis; miR-126 inhibition attenuated effects
miR-222 (Zhou et al., 2025)	Exosomes from SHED aggregates	NR	Activates angiogenesis-related pathways; promotes PDLSC angio-/osteo-differentiation	Rat alveolar bone defect/periodontal regeneration (PDLSC + exosomes)	Dose-dependent; regimen NR	↑New bone volume; ↑collagen deposition; ↑CD31^+^ microvessels
miR-21 (Wu D et al., 2022)	BMSC-sEV encapsulated in chitosan/β-GP thermosensitive hydrogel	*SPRY2*↓	Pro-angiogenic signaling activation; coupled osteogenesis	Rat critical-sized calvarial defect (5 mm)	Hydrogel containing sEVs: 200 μg (local implantation; volume NR)	↑BV/TV, BMD, Tb.N; ↑CD31^+^ microvessel density; ↑VEGF/bFGF/ANG1*etc.*
miR-26a (Kuang et al., 2023)	Exo@miR-26a–GelMA hydrogel (delivery system)	NR	Enriches osteogenesis/tissue remodeling signaling; suppresses osteoclast-related genes	Bone defect repair model (NR)	NR (local hydrogel delivery)	↑RUNX2, OPN, COL-I, BMP-2, *etc.*,; enhanced bone regeneration
miR-130a (Liu L et al., 2019)	BMSC exosomes induced by lithium-containing biomaterials	PTEN protein↓	↑p-AKT; ↑VEGF/ANG1/KDR/PDGF/HIF-1α, etc.	Implanted Li-BGC hollow scaffold (Microfil perfusion; vascular markers)	Li-BGC scaffold implantation (material specs/dose NR); exosome dose NR	↑Vessel ingrowth (Microfil; CD31/vWF/VEGF/ANG1, etc.)
miR-21 (An et al., 2019)	Engineered ADSC exosomes (miR-21 overexpression)	*PTEN*↓	↑AKT/ERK phosphorylation; ↑HIF-1α/VEGF/SDF-1	HUVEC tube formation *in vitro* (no bone model reported)	*In vitro*: 5 μg/mL exosomes (tube formation; 8–24 h)	↑Tube formation; ↑pro-angiogenic factors
miR-132 & miR-146a (Heo and Kim, 2022)	Human adipose MSC-derived exosomes (non-engineered)	Pro-angiogenic mediators miR-132/miR-146a; anti-inflammatory ROCK1/PTEN	Dual immunomodulatory and pro-angiogenic effects	THP-1 inflammation model; HUVEC proliferation/tube formation (no bone model)	*In vitro*: exosomes 5 μg/mL (e.g., 24 h)	↓TNF-α/IL-6/IL-8; ↑CD163/ARG1/CD206/IL-10, *etc.*,; ↑HUVEC proliferation and tube formation
miR-126 (Zhang L et al., 2021)	MSC exosomes from miR-126-overexpressing cells (engineered)	*PIK3R2*↓	PI3K/Akt activation	HUVEC migration/proliferation/tube formation (*in vivo* NR)	NR	↑Migration/proliferation/tube formation; ↑VEGF, Ang-1
miR-26a (Zuo et al., 2019)	CD34^+^ stem cell-derived exosomes (miR-26a transfected)	NR	Coupled angiogenesis and osteogenesis (GC-ONFH context)	Glucocorticoid-induced osteonecrosis of the femoral head (rat)	Exosomes: 100 μg in 200 μL PBS (route/frequency NR)	Reversed GC-impaired HUVEC migration/tube formation; ↑collagen deposition and bone reconstruction
miR-1260a (Wu et al., 2021)	BMSC exosomes after Fe_3_O_4_ nanoparticles + static magnetic field preconditioning	Osteogenic: *HDAC7*↓; Endothelial: *COL4A2*↓	Synchronous activation of osteogenic and angiogenic signaling	Mouse calvarial defect (5 mm; Gelfoam delivery)	Local implantation: Gelfoam with exosomes 200 μg in 100 μL PBS (single dose)	↑BMD, BV/TV; ↑CD31/α-SMA double-positive microvessels
let-7f-5p & miR-210-3p (Liu et al., 2022)	Hypoxia-conditioned SHED exosomes (2% O_2_; Hypo-exos)	*AGO1*↓ (let-7f-5p); *EFNA3*↓ (miR-210–3p)	↑VEGF signaling; removes anti-angiogenic cues	HUVEC proliferation/migration/tube formation; miRNA gain/loss assays (no defect model reported)	NR (primarily exosome treatment/miRNA modulation in HUVECs)	↑Tube formation and CD31^+^ lumen structures; hypoxia linked to ↑HIF-1α and *Rab27a*
miR-210-3p (Zhuang et al., 2022)	Hypoxia-conditioned MSC sEVs (1% O_2_)	*EFNA3*↓	miR-210–3p/EFNA3/PI3K axis	Calvarial defect; promotes type-H vessels (CD31^+^/Emcn^+^)	NR	↑BV/TV and vessel volume; ↑type-H vasculature

Gene symbols are used when referring to transcript-level regulation/miRNA targets, whereas protein nomenclature is used when protein cargos or protein-level changes are discussed EV, extracellular vesicles; sEV, small extracellular vesicles; MSC, mesenchymal stem/stromal cell; BMSC, bone marrow mesenchymal stromal/stem cell; ADSC, adipose-derived stromal/stem cell; EPC, endothelial progenitor cell; HUVEC, human umbilical vein endothelial cell; SHED, stem cells from human exfoliated deciduous teeth; PDLSC, periodontal ligament stem cell; PBS, phosphate-buffered saline; micro-CT, micro-computed tomography; BV/TV, bone volume/total volume; BMD, bone mineral density; Tb.N, trabecular number; IHC, immunohistochemistry; GC-ONFH, glucocorticoid-induced osteonecrosis of the femoral head; LAD, left anterior descending coronary artery; β-GP, β-glycerophosphate; GelMA, gelatin methacryloyl; NR, not reported.

Multiple mechanistic studies show that key miRNAs packaged in stem cell–derived EVs can promote angiogenesis and osteogenesis by targeting anti-angiogenic factors and thereby unleashing pro-vascular signaling. For instance, EVs from bone marrow mesenchymal stromal/stem cells (BMSCs) are enriched in miR-29a, which can be transferred to endothelial cells to suppress the anti-angiogenic factor *VASH1*, markedly enhancing endothelial tube formation *in vitro*. In animal models, miR-29a–loaded exosomes increased trabecular bone volume fraction (BV/TV), bone mineral density (BMD), and trabecular number (Tb.N), while also elevating neovessel density—supporting a dual pro-angiogenic and pro-osteogenic effect (Lu et al., 2020) Similarly, adipose-derived MSC (ADSC) exosomes carry abundant miR-21–5p, which enhances the angiogenic capacity of endothelial progenitor cells (EPCs) by repressing the NOTCH1/DLL4 axis and increasing *VEGFA* mRNA expression, thereby accelerating bone regeneration; *in vivo* administration of miR-21-5p–containing exosomes significantly improved both bone mass and vascular indices (Cao et al., 2024). In addition, miR-125a in human adipose-derived MSC exosomes directly targets *DLL4*, increasing the proportion of vascular tip cells (from ∼11% to ∼22%), alleviating Notch–DLL4–mediated constraints on sprouting angiogenesis, and enhancing vascular formation in HUVEC tube formation and Matrigel angiogenesis assays (Liang et al., 2016).

Beyond these examples, MSC exosomal miR-132 has also been shown to exert potent pro-angiogenic activity. After uptake by endothelial cells, miR-132 is upregulated and suppresses *RASA1*, thereby enhancing tube formation *in vitro*; in a myocardial infarction model, local injection of miR-132–enriched MSC exosomes increased capillary density and improved cardiac function (Ma et al., 2018). Exosomes secreted by EPCs depend on miR-126 to drive angiogenesis: miR-126 activates Raf/ERK signaling by inhibiting *SPRED1*, upregulating *HIF-1α*, *VEGF*, *TGF-β1*, and angiogenic factors such as *ANG*, which collectively enhance endothelial migration, proliferation, and tubulogenesis. In distraction osteogenesis models, local application of EPC exosomes increased osteogenic readouts and vascular volume fraction, whereas miR-126 inhibition blunted these pro-angiogenic and pro-osteogenic effects (Jia et al., 2019). Similarly, exosomes derived from stem cell aggregates of human exfoliated deciduous teeth (*SHED*) are enriched in miR-222, which dose-dependently promotes periodontal ligament stem cell (PDLSC) vasculogenic differentiation (increased network length and branching) and osteogenic differentiation (enhanced ALP activity and mineralization), alongside greater ECM deposition and activation of vascular-related programs. In a rat alveolar bone defect model, combined treatment with PDLSCs plus SHED exosomes yielded higher new bone volume, more collagen deposition, and increased CD31^+^ microvessel density compared with controls (Zhou et al., 2025).

In addition to direct administration of exosomes to stimulate angiogenesis and osteogenesis, regenerative medicine has increasingly leveraged biomaterial-assisted sustained release systems to prolong local exposure and improve therapeutic performance.

Conceptually, packaging miRNAs into EVs and then integrating EVs into scaffolds differs from directly immobilizing naked miRNA mimics on biomaterials (Wu D. et al., 2022). EV membranes protect encapsulated RNAs from extracellular RNases and provide a biocompatible nanoscale interface that supports cellular uptake and functional cytosolic delivery (Koga et al., 2011). Moreover, EVs co-deliver synergistic proteins and lipids that can prime recipient endothelial and immune cells, which may help explain why EV-based delivery often produces broader pro-angiogenic and immunoregulatory effects than single-cargo formulations (Gao et al., 2022). When EVs are embedded in hydrogels or porous scaffolds, the depot further increases local retention and enables sustained, stage-matched exposure—reducing the burst clearance seen after bolus dosing and improving spatiotemporal control of vascular cues (Hu et al., 2025). By contrast, direct miRNA loading onto biomaterials typically requires synthetic transfection carriers or chemical stabilization to preserve bioactivity, may suffer from rapid degradation or burst release, and lacks the multimodal signaling context intrinsic to EVs (Peng et al., 2015). Accordingly, EV-based miRNA delivery combined with biomaterial depots represents a more biomimetic and potentially translatable strategy for programming angiogenesis in bone defects.

Di et al. encapsulated BMSC-derived sEVs in a chitosan/β-glycerophosphate thermosensitive hydrogel, creating an injectable, *in situ* gelling, cell-free sustained-release platform. *In vitro*, this composite system dose-dependently promoted BMSC osteogenic differentiation and markedly enhanced endothelial angiogenic activities (migration, proliferation, and tube formation), accompanied by upregulation of pro-angiogenic factors such as VEGF, bFGF, and ANG1. In a rat critical-size calvarial defect model, implantation of the hydrogel–sEV construct significantly increased BV/TV, BMD, and Tb.N and elevated the density of CD31^+^ microvessels. Mechanistically, sEV-enriched miR-21 promoted angiogenic signaling by targeting and suppressing *SPRY2* in endothelial cells, thereby coordinately supporting bone regeneration (Wu D. et al., 2022). In a related approach, another study combined BMSC exosomes, miR-26a, and a gelatin methacryloyl (GelMA) hydrogel to build an efficient miRNA delivery system (“Exo@miR-26a-GC”), which synergistically upregulated osteogenesis-associated genes (*RUNX2*, *OPN*, *COL-I*, *BMP-2*). Transcriptomic analyses suggested that exosomes alone primarily activated angiogenesis, ECM remodeling, and cell migration pathways, whereas miR-26a loading further enriched osteogenic and tissue-remodeling programs and jointly suppressed osteoclast-related gene signatures (Kuang et al., 2023).

Notably, biomaterials can also act upstream to program exosomal miRNA loading, thereby amplifying angiogenic outputs. Lithium-doped bioceramics, as a chemical stimulus, were shown to activate AKT in BMSCs and co-activate Wnt/β-catenin and NF-κB pathways, enhancing endothelial migration and tubulogenesis. Importantly, this material selectively induced BMSCs to enrich miR-130a into exosomes and deliver it to endothelial cells. Exosome-mediated miR-130a transfer reduced endothelial PTEN protein (without changing *PTEN* mRNA), increased p-AKT activity, and broadly elevated angiogenic mediators including *VEGF*, *ANG1*, *KDR*, *PDGF*, and *HIF-1α*; inhibiting miR-130a in donor cells substantially weakened these effects. A 3D-printed lithium-doped bioactive glass–ceramic (Li-BGC) hollow scaffold further demonstrated robust vascular ingrowth *in vivo*, supported by Microfil perfusion imaging and molecular markers such as CD31, vWF, VEGF, and ANG1 (Liu L. et al., 2019).

From an engineering standpoint, genetic regulation, immunomodulation, and physical preconditioning have been widely used to re-shape exosomal miRNA cargo, strengthening the synergy between angiogenesis and bone regeneration. Using gene engineering, investigators produced miR-21–enriched exosomes from miR-21–overexpressing ADSCs, which significantly enhanced HUVEC tube formation. Mechanistically, these exosomes induced *PTEN* downregulation and increased AKT/ERK phosphorylation in both donor and recipient contexts, alongside upregulation of HIF-1α, VEGF, and SDF-1. This work linked overexpression of an oncogenic miRNA with EV engineering and provided experimental support for “programmable exosomal miRNAs” in vascularized regeneration (An et al., 2019).

Even without genetic modification, human adipose-derived MSC exosomes can exhibit dual anti-inflammatory and pro-angiogenic activity. In a THP-1 macrophage inflammation model, these exosomes reduced inflammatory cytokines (TNF-α, IL-6, IL-8) and upregulated anti-inflammatory markers (*CD163*, *ARG1*, *CD206,* TGF-β1, IL-10); the effect depended on activation of the ROCK1/PTEN axis, and blockade of this pathway diminished the anti-inflammatory response. In parallel, the same exosomes promoted HUVEC proliferation and tubulogenesis, upregulating *ANGPT1* and *KDR* while downregulating *THBS1* and *VASH1*. Further analyses implicated miR-132 and miR-146a as key mediators: both were markedly upregulated in recipient endothelial cells, and their inhibition reversed molecular changes and suppressed angiogenesis; dual inhibition almost completely abrogated tube formation (Heo and Kim, 2022).

Preconditioning stem cells via miRNA transfection can also enhance the pro-angiogenic and pro-osteogenic effects of exosomes. Enriching BMSC exosomes with miR-126 led to specific suppression of *PIK3R2*, activation of PI3K/Akt signaling, and increased HUVEC migration, proliferation, and tube formation, accompanied by elevated VEGF and Ang-1 expression (Zhang L. et al., 2021). Likewise, in glucocorticoid-induced osteonecrosis of the femoral head (GC-ONFH), CD34^+^ stem cell–derived exosomes—used as a naturally pro-angiogenic carrier—were further empowered by miR-26a transfection to strengthen osteogenic capacity. These exosomes reversed glucocorticoid-impaired HUVEC migration and tubulogenesis *in vitro* and improved collagen deposition and structural bone reconstruction in GC-ONFH rat models (Zuo et al., 2019).

Physical preconditioning (e.g., magnetic stimulation and hypoxia) can similarly reconfigure exosomal miRNA landscapes to enhance coupled angiogenesis and osteogenesis. Exosomes from BMSCs pretreated with magnetic Fe_3_O_4_ nanoparticles (50 μg/mL) plus a static magnetic field (100 mT) were enriched in miR-1260a, which promoted BMSC osteogenic differentiation and enhanced HUVEC migration and tubulogenesis *in vitro*. In a mouse calvarial defect model, a single implantation improved BMD and BV/TV and increased CD31^+^/α-SMA^+^ microvessels. Mechanistically, miR-1260a coordinately activated osteogenic and angiogenic programs by targeting *HDAC7* in osteoblast-lineage cells and *COL4A2* in endothelial cells (Wu et al., 2021).

Hypoxic preconditioning has likewise been shown to remodel exosomal miRNA profiles and amplify vascularized osteogenesis. Exosomes derived from SHED cultured at 2% O_2_ (“Hypo-exos”) more strongly promoted HUVEC proliferation, migration, and tube formation than normoxic controls and increased CD31^+^ luminal structures and VEGF signaling. Mechanistic analyses suggested that hypoxia-enriched let-7f-5p increases VEGF by suppressing *AGO1*, whereas miR-210–3p relieves angiogenic inhibition by targeting EphrinA3, with cooperative enhancement of vascular formation. Hypoxia also increased HIF-1α and the exosome secretion regulator *Rab27a* in donor cells, implying that hypoxia affects both miRNA loading and vesicle release (Liu et al., 2022). Consistently, exosomes secreted by MSCs preconditioned at 1% O_2_ enhanced endothelial angiogenic functions *in vitro* and increased BV/TV and neovessel volume in calvarial defect models, while also promoting formation of bone-associated H-type vessels (CD31^+^/Emcn^+^). Mechanistically, hypoxia increased exosomal miR-210–3p, which entered recipient cells and activated the PI3K/AKT axis by directly suppressing *EFNA3*; miR-210–3p knockdown diminished the pro-angiogenic/pro-osteogenic effects, whereas *EFNA3* inhibition partially rescued these phenotypes (Zhuang et al., 2022).

## 5 Engineering and targeted delivery strategies for EVs

As natural nanoscale carriers, extracellular vesicles (EVs) often exhibit limited intrinsic targeting after systemic administration. Following intravenous injection, many unmodified EV preparations show rapid clearance dominated by uptake within the mononuclear phagocyte system (MPS), with substantial accumulation in reticuloendothelial organs such as the liver and spleen. Consequently, achieving therapeutically meaningful enrichment at bone defects typically requires delivery strategies that increase local concentration, residence time, and bioactivity.

A shared objective across delivery designs is to increase local EV concentration, residence time, and biological activity at therapeutic sites such as bone defects. Current strategies broadly include: (1) biomaterial-enabled local and controlled release, and (2) EV surface engineering to enable active targeting. In addition, emerging approaches—including magnetic guidance, light-triggered release, and hybridization with nanocarriers—have shown promise. Below, we discuss these strategies and, from a translational perspective, highlight their safety considerations and scalability (Table 2).

**TABLE 2:** Comparison of engineering and targeted delivery strategies for EVs.

Delivery strategy	Advantages	Limitations/Risks	Typical use scenarios	Clinical translation potential
4.1 Hydrogel local controlled release (Lu et al., 2024; Wang L et al., 2020)	ECM-like 3D network; injectable/shape-conformal; protects exosomes and prolongs local retention; tunable release	Crosslinking/sterilization/solvents may impair bioactivity; limited mechanics for load-bearing sites; degradation–healing matching and burst release need optimization	Irregular craniofacial/alveolar defects; focal lesions requiring sustained local dosing (e.g., osteonecrosis)	Moderate–High: familiar biomaterials, but a combination product; requires GMP, sterility/shelf-life validation and release QC.
4.1 Porous/3D-printed scaffold loading (Turnbull et al., 2018; Sun et al., 2025b)	Provides mechanical support and an osteoconductive template; porosity enables adsorption and sustained release; patient-specific design	Implantation is required; loading homogeneity and release reproducibility depend on processing; higher cost and combination-device regulatory burden	Critical-size defects needing structural support (craniofacial, long bone, etc.)	Moderate: scaffold materials may be clinically established, but exosome functionalization needs standardized loading/sterilization and release testing
4.1Electrospun membranes/biomimetic periosteum (Berton et al., 2019; Yun et al., 2024)	High surface area and ECM-like fibrous architecture; stable immobilization with sustained release; can serve as GBR membrane	Still limited evidence in bone regeneration; handling/fixation and mechanics need optimization; solvent-related risks if processing is not well controlled	Barrier membranes for oral/periodontal GBR; soft–hard tissue interfaces	Moderate: membrane materials are mature; EV loading, sterility and shelf-life remain to be validated
4.1 Injectable microsphere/particle carriers (Gao et al., 2022)	Minimally invasive injection; controllable release kinetics; can create pro-angiogenic microenvironments (e.g., sustained release under hypoxic cues)	Manufacturing complexity (size distribution, encapsulation efficiency); potential polymer-related inflammation; hard to retrieve after injection	Irregular/deep defects where injection is preferred; minimally invasive adjunct delivery	Moderate–Low: scalable in principle, but reproducibility, potency assays and regulatory pathway for EV-loaded particles remain challenging
4.1 Bioactive ceramics/bioactive glass functionalization (Ranmuthu et al., 2020)	Inherent osteoconductive/osteostimulative properties; surface functionalization adds pro-angiogenic and osteogenic cues	Brittleness/limited toughness; surface adsorption may cause burst release; sterilization/storage can affect EV potency	Non-load-bearing defects; craniofacial applications; fillers/coatings	Moderate: familiar materials, but consistent EV coating and potency/QC assays are required
4.1 Endogenous exosome-capturing scaffold (Wang L et al., 2025)	No exogenous EV production; enriches host-derived EVs at the defect site; leverages innate repair for fast vascularization	Depends on host inflammatory/exosome response; coating specificity and safety must be validated; mechanism may vary across conditions and individuals	Implantable defects with intact endogenous repair potential; when exogenous EV supply is limiting	Moderate: avoids EV supply chain but needs robust cross-model efficacy and well-defined manufacturing of the capture coating
4.1 Biodegradable metal scaffold + exosomes (Zhang et al., 2024)	Load-bearing support; biodegradation can release bioactive ions; EVs add angiogenic/osteogenic cues	Degradation rate and ion safety need control; complex combination product; long-term biocompatibility and corrosion products must be evaluated	Segmental defects requiring mechanical support (preclinical large defects)	Moderate: promising for orthopedics, but regulatory/CMC and long-term safety are major hurdles
4.2 Covalent chemical conjugation (Musicò et al., 2023; Zhang H et al., 2019; Zhao Z et al., 2022)	Fast and efficient bioorthogonal reactions; controllable ligand density; stable display of peptides/antibody fragments	Requires removal of residual reagents; may alter the protein corona and cellular uptake; added ligands may increase immunogenicity—needs rigorous evaluation	Systemic dosing where higher tissue selectivity is needed; diseases with validated surface targets (e.g., neovasculature/inflamed endothelium)	Moderate: scalable chemistry, but a complex modified EV product—stringent CMC and immunosafety packages required
4.2 Bone-homing anchoring: bisphosphonate/ALN lipid insertion (Zheng et al., 2022)	High affinity to bone mineral; increases bone accumulation after IV administration; may enhance bone–vessel coupling and osteogenesis	PEG/ALN can affect uptake and immune interactions; off-target deposition and long-term toxicity must be assessed; requires robust PK profiling	Systemic bone diseases (osteoporosis/osteonecrosis/multifocal lesions); enhancing peri-defect bone quality and vascularization	Moderate–High: bone-homing moieties are established, but EV–lipid insertion is a complex nanoformulation requiring stringent PK/toxicology and QC.
4.2 Genetic engineering surface display (Alvarez-Erviti et al., 2011; Antes et al., 2018; Xu et al., 2021; Hung and Leonard, 2015)	Modification occurs in producer cells, giving better consistency; oriented display (e.g., RVG, E7) without chemical residues; compatible with repeated dosing	Time-consuming cell line development: fusion proteins may reduce EV yield or be degraded; more complex regulatory expectations (cell bank, genetic stability, viral safety)	High specificity targeting (CNS, specific cell subsets such as MSCs); systemic delivery with stringent cell selectivity	Moderate: strong academic foundation, but CMC/cell bank and batch consistency are key barriers
4.2 Affinity/noncovalent anchoring (Liu et al., 2023)	Mild conditions; modular “plug-and-play” ligand screening; avoids harsh covalent chemistry	Lower *in vivo* stability (ligand shedding/exchange); potential immunogenicity (e.g., streptavidin); aggregation risk	Early-stage proof-of-concept and rapid ligand screening; local/short-course dosing where long-term stability is less critical	Low–Moderate: useful for discovery, but clinical products often prefer more stable covalent or genetic approaches
4.2 PEGylation stealth + terminal targeting (Kooijmans et al., 2016)	Reduces MPS clearance and prolongs circulation; PEG termini can present targeting ligands to improve specificity	May hinder cellular uptake; anti-PEG antibodies/complement activation concerns; complex characterization and QC.	IV systemic dosing to reduce liver/spleen sequestration; can be combined with other targeting strategies	Moderate: extensive nanomedicine precedent, but EV-specific repeated-dosing immunology needs more data
4.2 Hybrid exosomes (Sato et al., 2016)	Combines EV biocompatibility with high loading and design flexibility of synthetic carriers; can improve loading efficiency and stability	More complex manufacturing and characterization; batch heterogeneity; altered membrane proteome may change immunology/targeting; regulatory path uncertain	Systemic delivery requiring high payload (RNA/protein/small molecules) while aiming for EV-like biodistribution	Moderate–Low: attractive performance, but high CMC complexity and regulatory uncertainty
4.3 Magnetic field–guided targeting (Villa et al., 2024)	Externally controllable accumulation at the target site; can be integrated with imaging and other physical therapies	Safety and long-term fate of magnetic materials; limited field penetration and equipment dependence; higher operational complexity and cost	Lesions accessible to localized magnetic fields; perioperative assistance for bone defects or repeated dosing	Low–Moderate: niche opportunities, but device dependence and material safety are major hurdles
4.3 Stimuli-responsive spatiotemporal release (Liu Y et al., 2019; Rui et al., 2023)	On-demand release with spatiotemporal precision; reduced systemic exposure; supports staged immuno-regenerative modulation	Light has limited tissue penetration; ultrasound parameters/safety windows need optimization; often multi-component systems with complex QC.	Localized lesions amenable to external triggers; phased repair processes needing pulsatile dosing	Low–Moderate: largely preclinical; simplification and reproducibility are needed for translation
4.3 Exosome-membrane-camouflaged/mimicked nanocarriers (Poinsot et al., 2024; Lopes et al., 2023)	High loading with tunable size; EV membranes provide immune evasion and partial homing; potentially more scalable manufacturing	Not natural exosomes in a strict sense; membrane source and immunology must be controlled; complex product definition and regulatory classification	Systemic delivery requiring tight payload control (gene/protein combinations); exploring bone-targeted variants by adding bone-homing ligands	Moderate: can leverage nanomedicine experience but needs clear characterization standards and *in vivo* fate data
4.3 Integrated/combinatorial approaches (Liu et al., 2023)	Addresses both arrival and retention barriers; may reduce dose and prolong efficacy; enables synergy across angiogenesis, osteogenesis and immunomodulation	Multi-component systems increase manufacturing, release testing and regulatory complexity; large design space requires robust PK/tracing frameworks	Complex/large-volume defects; staged healing (vascularization first, then osteogenesis)	Moderate: high therapeutic promise, but relies on standardization, simplification and thorough safety evaluation

Clinical translation potential is a qualitative assessment (High/Moderate/Low) for review comparison; translation depends on GMP manufacturing, sterility/shelf-life validation, release QC and regulatory pathway. General EV product quality/safety considerations: (Takakura et al., 2024); industrial/clinical development trends: (Lee, 2024) chemistry, manufacturing, and controls (CMC); pharmacokinetics (PK); quality control (QC).

### 5.1 Biomaterial-based delivery

**FIGURE 5:**
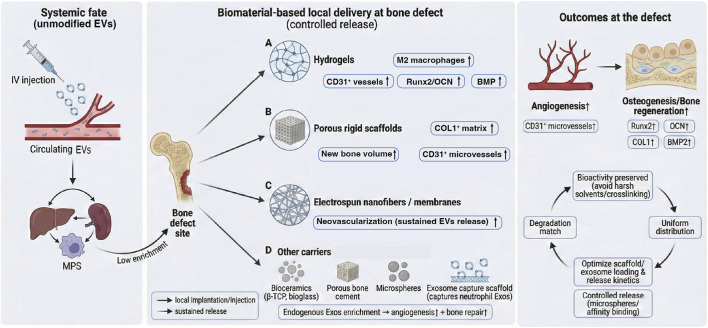
Biomaterial-based delivery strategies for EVs at bone defects. Left: systemic fate of unmodified EVs after intravenous (IV) injection. Circulating EVs are predominantly cleared/sequestered by the mononuclear phagocyte system (MPS; e.g., liver/spleen) with low enrichment at the bone defect. Middle: biomaterial depots for local delivery enable controlled and sustained EV release at the defect site, including **(A)** injectable hydrogels, **(B)** porous rigid scaffolds, **(C)** electrospun nanofibers/membranes, and **(D)** other carriers (bioceramics such as β-tricalcium phosphate (β-TCP) and bioglass, porous bone cement, microspheres, and EV-capture scaffolds to enrich endogenous EVs). Right: typical outcomes and design considerations. Local EV depots enhance angiogenesis (e.g., increased CD31^+^ microvessels) and osteogenesis (e.g., RUNX2, OCN, COL1 and BMP2 upregulation) while emphasizing preservation of EV bioactivity, matching carrier degradation to healing kinetics, achieving uniform distribution, and tuning loading/release kinetics (e.g., affinity binding or microsphere encapsulation) to reduce burst clearance. Created by the authors based on representative biomaterial-delivery and bone-regeneration literature (Yan et al., 2020; Kang et al., 2021; Wu et al., 2022; Gao et al., 2022; Witwer and Wolfram, 2021). Abbreviations: β-TCP, beta-tricalcium phosphate; BMP2, bone morphogenetic protein 2; COL1, type I collagen; EV, extracellular vesicle; IV, intravenous; MPS, mononuclear phagocyte system; OCN, osteocalcin; RUNX2, runt-related transcription factor 2.

Biomaterials (e.g., collagen sponges, hydrogels, electrospun nanofibers, and porous scaffolds) can be used to load EVs and implant or inject them into the target site, enabling spatially localized release. Compared with direct injection of free EVs, scaffold-based EV delivery typically provides prolonged local exposure, higher effective concentrations, and essential tissue-engineering support (Figure 5).

#### 5.1.1 Hydrogels as EV depots

Hydrogels, with a three-dimensional network and high water content reminiscent of extracellular matrix, serve as attractive EV carriers (Lu et al., 2024). Incorporating EVs into hydrogels can markedly extend residence time and improve stability at injury sites (Yang et al., 2020). For example, periodontal ligament stem cell–derived exosomes embedded in a gelatin–sodium alginate hydrogel and implanted into alveolar bone defects significantly enhanced neovascularization and bone regeneration (Zhao Y. et al., 2022). In a glucocorticoid-induced osteonecrosis of the femoral head model, Chen et al. encapsulated lithium-preconditioned BMSC exosomes (Li-Exos) within a type I collagen methacrylate hydrogel, which increased CD31^+^ vessel density in ischemic regions, elevated osteogenic markers such as *RUNX2* and *OCN*, and promoted M2 macrophage infiltration—collectively improving bone regeneration (Chen C. et al., 2023). In addition, a newly developed self-healing hydrogel composed of coral acid hydroxyapatite (CHA)/silk fibroin (SF)/polyethylene glycol (PEG)-chitosan (GCS)/dysfunctional PEG (DF-PEG), when loaded with umbilical cord MSC exosomes, showed strong pro-angiogenic and osteogenic activity, with increased new bone formation, upregulated local BMP2, and significantly elevated microvessel density (Wang L. et al., 2020).

#### 5.1.2 Porous scaffolds and structural carriers

Functionally integrating EVs into rigid scaffolds is another key route to enhance bone repair. Porous scaffolds (including 3D-printed scaffolds, bioceramics, and porous metals) provide mechanical support and a structural template for bone ingrowth, while their porosity facilitates EV adhesion and sustained release (Sun et al., 2025b; Turnbull et al., 2018). Sun et al. fabricated a silk fibroin/collagen/nano-hydroxyapatite composite scaffold using low-temperature 3D printing and loaded it with human umbilical cord MSC exosomes for a severe alveolar defect model in rats. Compared with the scaffold-only group, the “scaffold + EV” group showed a substantially higher fraction of new bone volume (∼62% vs. ∼42%) and significantly increased neovessel numbers within the defect. Histology and immunostaining further revealed enhanced COL1^+^ new bone matrix deposition and denser CD31^+^ microvessels with larger luminal diameters, indicating improved vessel maturity and functional perfusion (Sun et al., 2023).

#### 5.1.3 Electrospun membranes and interfacial constructs

Electrospun membranes feature high surface area and biomimetic fibrous architecture and can serve as guided bone regeneration (GBR) membranes or soft–hard tissue interface scaffolds (Berton et al., 2019). EVs can be adsorbed onto or embedded within electrospun nanofibers to achieve stable immobilization and sustained release. One approach impregnated a 3D-printed calcium silicate bioceramic scaffold with a polycaprolactone (PCL) solution and electrostatically adsorbed EVs, yielding a platform capable of sustained EV release. EVs released from the scaffold surface markedly promoted neovessel formation around the implant and accelerated vascularized bone repair (Yun et al., 2024; Su et al., 2022). Although reports on electrospun EV delivery in bone regeneration remain limited, the tunable surface properties of membrane materials make this strategy attractive for clinical translation.

#### 5.1.4 Bioceramics, bone cements, microspheres, and “EV-capture” scaffolds

Bioceramics, porous bone cements, and microspheres have also been employed for EV delivery. Bioactive ceramics such as β-tricalcium phosphate (β-TCP) and bioglass inherently support bone ingrowth; after surface functionalization and EV loading, they can further enhance angiogenic and osteogenic potency (Zhang J. et al., 2016; Gao et al., 2022; Ranmuthu et al., 2020). For instance, Qi et al. loaded hiPSC-MSC exosomes (hiPSC-MSC-Exos) onto β-TCP porous scaffolds for critical-size calvarial defects in ovariectomized rats, and observed significantly increased neovessel formation and new bone volume compared with controls. More recently, an “exosome-capture scaffold” strategy has been proposed: specialized coatings are used to capture endogenous exosomes secreted by host neutrophils, enriching local EVs and producing notable pro-angiogenic and pro-osteogenic effects (Wang L. et al., 2025). Importantly, this approach does not require exogenous EV supplementation and instead leverages intrinsic repair mechanisms, showing strong vascularized bone regeneration in animal models.

#### 5.1.5 Engineering challenges and translational considerations

Despite clear advantages, biomaterial-based EV delivery faces several engineering challenges. First, preserving EV bioactivity during scaffold fabrication is critical, necessitating avoidance of organic solvents or crosslinking conditions that may compromise EV structure and function. Second, scaffold degradation should be matched to the tempo of bone regeneration: overly slow degradation may impede new bone ingrowth, whereas overly rapid degradation may trigger premature EV burst release and reduce durability of benefit. Third, uniform EV distribution within scaffolds and controllable release require careful design; approaches such as microsphere intermediates or affinity-binding strategies may help stably immobilize EVs within scaffold architectures.

From a translational standpoint, scaffold materials for EV delivery should ideally be clinically approved implantable materials, such as collagen and polylactic acid (PLA). Manufacturing must comply with Good Manufacturing Practice (GMP) standards, and sterilization and storage conditions must preserve EV bioactivity. Notably, some groups have begun moving toward large-animal studies and preclinical prototypes. For example, a 3D-printed biodegradable porous zinc scaffold loaded with EVs achieved effective repair in a rabbit segmental bone defect model, providing early evidence of translational potential (Zhang et al., 2024).

Overall, biomaterial-enabled local EV delivery provides an efficient, cell-free modality for bone regeneration. Future efforts should optimize loading efficiency, release kinetics, and the breadth of safety/efficacy datasets to accelerate clinical translation.

### 5.2 Surface engineering and targeting modifications

**FIGURE 6:**
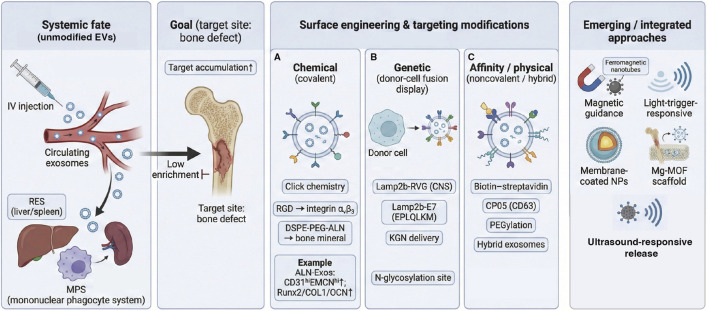
Surface engineering and targeting modifications of EVs. **(A)** Chemical conjugation (post-isolation modification): purified EVs are functionalized by covalent chemistries (e.g., copper-free click chemistry) or by hydrophobic insertion of amphiphiles (e.g., DSPE-PEG-ALN, where ALN is alendronate) to introduce bone-mineral affinity and/or endothelial targeting ligands (e.g., integrin αvβ3-binding motifs). **(B)** Genetic engineering (donor-cell modification): producer cells are transfected to express ligand/peptide fusions with EV membrane anchors (e.g., Lamp2b–ligand fusion proteins), resulting in EVs with enhanced uptake by defined target cells (e.g., synovial MSCs or bone marrow cells). **(C)** Physical, affinity and emerging strategies: affinity anchoring (e.g., biotin–avidin), magnetic guidance under an external magnetic field, and hybrid/coating approaches such as MOF–EV scaffolds to improve local accumulation and regenerative efficacy. Created by the authors based on representative EV engineering and targeting studies (Alvarez-Erviti et al., 2011; Liang et al., 2021; Zheng et al., 2022; Kang et al., 2022; Villa et al., 2024; Wang et al., 2023). Abbreviations: ALN, alendronate; DSPE-PEG, 1,2-distearoyl-sn-glycero-3-phosphoethanolamine–poly (ethylene glycol); EV, extracellular vesicle; Lamp2b, lysosome-associated membrane glycoprotein 2b; MOF, metal–organic framework; MSC, mesenchymal stromal/stem cell.

Engineering the EV surface to confer active targeting has become a central strategy to improve delivery efficiency. Current surface-engineering approaches mainly include chemical conjugation, genetic engineering of donor cells, and affinity-based anchoring. Conceptually, these methods introduce targeting ligands (e.g., small molecules, peptides, or antibody fragments) onto the EV membrane, enabling EVs to actively recognize and preferentially accumulate in target tissues. Compared with passive “homing” mediated by native EV membrane proteins, active targeting can substantially increase selectivity and tissue accumulation at the intended site (Figure 6).

#### 5.2.1 Chemical conjugation

Chemical conjugation uses bioorthogonal reactions to covalently attach targeting moieties to the membrane of purified EVs. Typical implementations include click chemistry, which enables rapid and efficient coupling through reactive functional groups on the EV surface (e.g., surface amines or terminal groups) while largely preserving membrane integrity (Smyth et al., 2014). The resulting modifications are generally stable *in vivo* and less prone to ligand dissociation (Musicò et al., 2023).

For example, the RGD tripeptide (Arg–Gly–Asp) can recognize integrins (e.g., αvβ3) on vascular endothelial cells, thereby improving EV enrichment and uptake in ischemic tissues (Zhang H et al., 2019). Although many RGD-display studies are performed in tumor neovasculature as a tractable *in vivo* model, the same ligand–integrin targeting logic is transferable to ischemic bone defects where endothelial integrins are upregulated during neovascularization (Zhao Z. et al., 2022). In addition, DSPE–PEG–alendronate (DSPE–PEG–ALN) can insert into the EV membrane to display alendronate (ALN), conferring high affinity for bone mineral (Zheng et al., 2022). In a glucocorticoid-induced osteoporosis model, Zheng et al. showed that ALN-modified platelet EVs accumulated more efficiently in bone, increased H-type vessels (CD31^hi^EMCN^hi^), and upregulated osteogenic markers including *RUNX2*, *COL1*, and *OCN*—supporting the therapeutic potential of bone-targeted EVs to promote bone–vascular coupling and regeneration (Zheng et al., 2022).

Overall, chemical conjugation offers practical advantages—simplicity, controllability, and scalability/standardization—but it requires rigorous removal of residual reagents and careful evaluation of potential immunogenicity introduced by the modification.

#### 5.2.2 Genetic engineering of donor cells

Genetic engineering operates at the level of donor cells, programming them to secrete EVs that display targeting ligands on their surface. A widely used strategy is the fusion-protein approach, in which a targeting peptide or antibody fragment is genetically fused to an EV-enriched membrane protein (e.g., Lamp2b) or other membrane-anchoring domains, leading to EVs that naturally present the ligand on their membrane (Alvarez-Erviti et al., 2011; Antes et al., 2018).

Since the seminal work by Alvarez-Erviti et al. demonstrating Lamp2b-mediated display of RVG for brain-targeted delivery, this platform has been broadly adopted across targeting applications (Alvarez-Erviti et al., 2011). For example, investigators engineered EVs by using Lamp2b to display an MSC-binding peptide E7 (sequence: EPLQLKM), generating E7-decorated EVs (E7-Exo) (Xu et al., 2021). These E7-Exo preferentially targeted MSC subpopulations in synovium or bone marrow, thereby improving delivery of the chondrogenic small molecule kartogenin (KGN) and enhancing chondrogenic differentiation.

Genetic approaches benefit from intracellular assembly and often yield higher modification uniformity across EV batches. However, establishing stable, high-producing engineered cell lines can be time-consuming, and fusion proteins may alter EV biogenesis or be susceptible to intracellular degradation. To enhance stability, N-terminal glycosylation motifs are often introduced into Lamp2b–peptide constructs to reduce proteolytic cleavage during secretion (Hung and Leonard, 2015).

#### 5.2.3 Affinity anchoring and other physical modification strategies

Emerging non-covalent and physical approaches are also gaining attention. One representative strategy exploits the high-affinity biotin–streptavidin interaction: EVs are first biotinylated, and streptavidin tetramers bearing targeting antibodies are then used to immobilize antibodies on the EV surface (Liu et al., 2023). Another method uses the CP05 peptide, which binds the EV marker CD63, as an anchoring handle to enable non-covalent attachment of targeting ligands (Gao et al., 2018). Hybridization strategies have also been proposed, including fusing EVs with targeted liposomes or cell-membrane vesicles to generate hybrid EVs that integrate complementary advantages of different carriers (Sato et al., 2016). PEGylation can improve circulation stability and can be used to display targeting ligands at PEG termini (Kooijmans et al., 2016).

A recurring limitation is that non-covalent modifications are often less stable *in vivo* than covalent linkages and may suffer from ligand dissociation or EV aggregation. In practice, combinations of methods are frequently required to achieve robust stability and functional targeting.

Across all modification strategies, stringent safety evaluation is essential—particularly regarding ligand-triggered immune responses (e.g., antibody formation or complement activation) and long-term foreign-body toxicity (e.g., residual polymers or nanomaterials) (Takakura et al., 2024). Manufacturing must also ensure consistency, reproducibility, and scalability. Chemical conjugation requires strict quality control (QC) of EV purification and coupling stoichiometry; genetic engineering requires stable, high-yield producer cell lines and tightly controlled downstream purification to avoid residual free ligands or carriers. In targeting studies, EVs are commonly labeled (fluorescent or radiotracer) to quantify biodistribution changes introduced by modifications and to iteratively optimize targeting designs.

### 5.3 Other emerging strategies and integrated approaches

Beyond the mainstream approaches above, innovative delivery strategies are being explored to enhance targeting efficiency and achieve spatiotemporally controlled EV delivery. One direction leverages physical fields or external stimuli to guide EV accumulation or trigger on-demand release. For example, magnetic targeting can be achieved by coupling EVs to superparamagnetic nanoparticles or magnetic carriers; after systemic administration, an external magnetic field can concentrate EVs at the target site. Villa et al. immobilized myogenic EVs on ferromagnetic nanotubes and, under an applied magnetic field, achieved efficient enrichment at skeletal muscle lesions after intravenous injection, accompanied by immunometabolic remodeling (Villa et al., 2024). In addition, light-triggered or ultrasound-responsive EV platforms have been developed to enable spatiotemporally controlled release in rheumatoid arthritis or tumor models (Liu Y. et al., 2019; Rui et al., 2023).

Another rapidly developing area is biomimetic EV-inspired systems, in which inorganic or polymeric nanoparticles serve as cores and EV membranes serve as shells—combining high payload capacity with immune stealth and target recognition (Poinsot et al., 2024; Lopes et al., 2023). For instance, microfluidic/ultrasonic methods have been used to coat polymeric nanoparticles with tumor cell–derived EV membranes to generate exosome membrane–coated nanoparticles, yielding enhanced immune evasion and tumor accumulation *in vivo*. Similarly, porous silicon nanoparticles coated with EV membranes have shown both high drug loading and EV-like homing, improving intratumoral drug enrichment and chemotherapy efficacy.

In bone tissue engineering, EVs have also been integrated with emerging carrier materials such as metal–organic frameworks (MOFs) (Kang et al., 2022). Because MOF–EV hybrids represent integrated carrier systems (material microenvironment modulation plus EV signaling) rather than pure surface targeting, we highlight them here as an emerging integrated approach in bone tissue engineering. EV-functionalized Mg-MOF scaffolds have reportedly enabled synergistic gains across osteogenesis, angiogenesis, and anti-inflammatory effects in cranial defect models (Kang et al., 2022). Notably, while bone-targeting moieties (e.g., bisphosphonates, Asp8, Asp–Ser–Ser) are widely used in liposomes, polymer micelles, and inorganic nanomaterials to improve deposition in bone and bone marrow, direct evidence in bone disease/defect models that combines EV membranes with bone-targeting nanostructures remains relatively limited and early-stage.

From a translational standpoint, engineered and targeted EVs have begun to take shape through industry efforts exemplified by platforms from companies such as Codiak, Evox, and ILIAS (Lee, 2024). As one illustrative example, Codiak’s engEx®-based approaches have explored surface display of PTGFRN-fused ligands and luminal loading of immune modulators (e.g., STING agonists/IL-12) for tumor-localized delivery, with Phase 1 studies providing early platform-level pharmacology and safety signals. Evox Therapeutics has used ExoEdit™-type strategies to package gene-editing tools and RNA therapeutics into engineered EVs for CNS-targeted delivery in rare neurological diseases. In South Korea, ILIAS Biologics has used EXPLOR®-based approaches to enable light-controlled protein loading and advanced candidates such as ILB-202 into early clinical evaluation, highlighting the translational potential of engineered EVs for inflammation-associated diseases (Lee, 2024).

In summary, EV targeting and delivery is a rapidly advancing Frontier. Whether via local, sustained release from biomaterial scaffolds, active targeting through surface engineering, or emerging stimulus-responsive and hybrid carrier systems, the overarching goal is to maximize EV concentration and duration of action at disease sites. Future work will likely benefit from integrated strategies—for example, combining scaffold-based local delivery with EV surface targeting, or pairing EV therapy with gene/drug/physical interventions for synergistic benefit. Critically, the field also needs standardized *in vivo* tracing and pharmacokinetic frameworks to more precisely map EV distribution, metabolism, and fate under different delivery designs, thereby enabling rational optimization. As EV biology and engineering mature, more efficient and safer targeted EV therapeutics are expected to emerge, with meaningful implications for clinical bone defect repair.

## 6 Key issues and current status in clinical translation

### 6.1 Challenges for the clinical translation of stem cell–derived EVs

#### 6.1.1 Scale-up manufacturing and purification

##### 6.1.1.1 Centrifugation-based methods

Differential ultracentrifugation (dUC) remains the most widely used foundational approach (Zheng et al., 2024). By sequentially increasing centrifugal forces to remove cells and organelles, small EVs are finally pelleted at ≥100,000 × g. Its key advantages are that the workflow is mature and does not introduce exogenous polymers or antibodies, making it particularly suitable for isolating medium-to-high purity MSC-EVs from relatively “clean” MSC-conditioned media. Major limitations include high equipment cost and labor/time intensity; prolonged exposure to high g-forces can deform vesicles and promote aggregation. In complex matrices such as plasma, co-precipitation of proteins and lipoproteins is common, making dUC poorly aligned with clinical-grade requirements for continuous processing and standardization. In practice, dUC is better suited to laboratory-scale preparation and is often combined with density gradients or SEC to improve purity.

Density gradient ultracentrifugation (sucrose or iodixanol gradients) separates particles by buoyant density, effectively removing abundant proteins and fractionating EV subpopulations—highly valuable for EV heterogeneity studies and for generating high-purity samples for proteomics/lipidomic (Jia et al., 2022). However, gradient preparation and fraction collection are operationally demanding, yields can be modest, and run times are long; thus, it is used primarily for analytical-grade preparations rather than routine production.

##### 6.1.1.2 Membrane filtration and tangential flow filtration

Conventional ultrafiltration (UF) uses membranes with defined molecular-weight cutoffs to retain EVs under pressure or centrifugation, enabling rapid concentration of large volumes of conditioned media or biofluids without polymer additives. UF is readily integrated with downstream chromatography (e.g., UF–SEC or ultrafiltration (UF)–anion-exchange chromatography (AEX)) (Li B. et al., 2025). Nonetheless, membrane adsorption and shear can cause EV loss or damage, and high-protein samples are prone to fouling—so UF is often best positioned as a pre-concentration step.

Tangential flow filtration (TFF) is a scalable, continuous form of UF in which fluid sweeps parallel to the membrane surface, reducing cake formation and enabling controlled-shear concentration and diafiltration. TFF has emerged as a core technology for clinical-scale MSC-EV production (Li M. et al., 2025). Its drawbacks are higher equipment/consumable costs and the need for downstream “polishing” (typically SEC and/or AEX) to achieve clinical-grade purity.

##### 6.1.1.3 Chromatography

Size-exclusion chromatography (SEC) separates particles by hydrodynamic radius; EVs elute earlier than soluble proteins (Kim et al., 2025). SEC runs at ambient conditions with minimal structural stress to vesicles and offers high reproducibility *via* commercial prepacked columns, making it widely used for EV purification from plasma, urine, and MSC-conditioned media. Limitations include restricted loading volumes and dilute fractions requiring reconcentration; SEC also has limited resolving power against similarly sized lipoproteins or protein aggregates. Consequently, SEC is frequently used after UF/TFF as a polishing step.

Anion-exchange chromatography (AEC) exploits the net negative charge of EV surfaces: vesicles bind to positively charged matrices and are eluted using salt gradients, enabling removal of charged impurities in protein/nucleic-acid–rich backgrounds. AEX is often combined with TFF or SEC as a two-dimensional purification strategy (Li B. et al., 2025). Its sensitivity to buffer composition and ionic strength—and the requirement for thorough desalting after high-salt elution—make AEX most suitable as a final polishing step for high-purity, clinically translatable MSC-EV products.

##### 6.1.1.4 Polymer co-precipitation

PEG–based co-precipitation leverages volume-exclusion effects to sediment EVs at low-speed centrifugation and underpins many commercial kits (Huang et al., 2025). It is simple, equipment-light, and yields high apparent recovery, making it useful for rapid, coarse enrichment from limited clinical samples or exploratory RNA profiling. However, PEG also co-precipitates soluble proteins, lipoproteins, and immune complexes, and residual PEG is difficult to remove—confounding proteomics and functional assays. Accordingly, MISEV guidance discourages its use as a sole method for quantitative/functional studies and it is generally unsuitable for clinical-grade manufacturing. In practice, PEG is sometimes used for initial enrichment when sample is scarce and RNA exploration is the main goal, followed by SEC/UC for further purification.

##### 6.1.1.5 Immunoaffinity capture and immunomagnetic beads

Immunoaffinity methods selectively capture EVs via surface tetraspanins (CD9/CD63/CD81) or MSC/disease-specific markers using antibody/aptamer-coated magnetic beads, chromatographic matrices, or chip surfaces. They can achieve very high purity and enrich specific EV subpopulations from μL–mL samples—particularly valuable for liquid biopsy and biomarker studies (Ljungström and Oltra, 2025). Limitations include high reagent/carrier cost, poor scalability, representation bias toward epitope-positive EV subsets, and potential damage to surface proteins/function during harsh elution (low pH/high salt). Thus, immunoaffinity capture is best suited to diagnostics and mechanism studies rather than large-scale MSC-EV production.

##### 6.1.1.6 Microfluidics

Microfluidic platforms precisely manipulate microliter-scale fluids to enable rapid EV separation/detection via physical principles (size, inertia, acoustics) or biochemical capture (immunoaffinity) (Wu Y. et al., 2022). Dual-membrane filtration chips, nanopore arrays, and acoustic/inertial sorters can isolate EVs from tens of microliters of plasma within <1 h; antibody/aptamer-functionalized channels can integrate capture–wash–release–detection, enabling “sample-in, answer-out” diagnostic systems when coupled to electrochemical/fluorescent readouts (Iliescu et al., 2019). Microfluidics excels in low sample requirements, speed, integration, and automation—well suited for precious samples such as cerebrospinal fluid. However, most devices remain at prototype stage, with limited throughput and relatively high fabrication costs; they are currently positioned for diagnostics/high-throughput screening rather than clinical-scale MSC-EV manufacturing.

##### 6.1.1.7 Ultrafast automation and emerging high-resolution tools

Automated ultrafast systems such as EXODUS combine nanoporous membranes with negative-pressure oscillation and dual-frequency vibration to enrich EVs from diverse biofluids within 30–60 min, achieving high recovery and protein-removal efficiency across a broad volume range. Disposable cartridges enhance reproducibility across centers, and EXODUS has already been applied to EV biomarker studies in Alzheimer’s disease, prostate cancer, and other conditions (Chen et al., 2021). However, instrument and consumable costs remain substantial; high-viscosity samples require preprocessing; and current use is mainly in diagnostics/biomarker discovery.

Asymmetric flow field-flow fractionation (AF4) applies a cross-flow field in an unpacked channel and fractionates particles by Brownian diffusion, offering high-resolution size separation with minimal shear and non-specific adsorption. AF4 can separate EVs from smaller nanoparticles (e.g., exomeres) and integrates well with MALS, NTA, and mass spectrometry for fine characterization and QC of clinical-grade preparations. Yet AF4 is expensive, method development is complex, and throughput is limited—making it primarily an advanced analytical tool rather than a routine production method (Zhang et al., 2018).

Overall, MSC-EV isolation involves clear trade-offs among purity, yield, sample requirements, scalability, and cost. For mechanistic studies, common workflows include dUC or UF/TFF preconcentration followed by SEC, with density gradients or AEX added when higher purity is required. Omics and quantitative studies are better served by UF/TFF–SEC or UF/TFF–SEC–AF4, avoiding PEG as a stand-alone approach. Liquid biopsy and diagnostic applications prioritize specificity and sensitivity, where immunoaffinity capture or microfluidic immune chips can serve as core platforms, optionally complemented by high-recovery systems such as EXODUS. For clinical-grade formulations and large-scale manufacturing, the prevailing consensus is TFF as the backbone for concentration/diafiltration, followed by SEC/AEX polishing, alongside GMP-compliant in-line/off-line QC. Across basic and translational settings, combining methods—rather than relying on a single technique—has become the pragmatic route for advancing MSC-EVs toward clinical use (Table 3).

**TABLE 3:** Comparison of EV isolation and preparation methods.

Method	Main principle/Typical combinations	Main advantages	Main limitations	Typical applications	Scale-up and clinical translation potential
Differential ultracentrifugation (dUC) (Zheng et al., 2024)	Sequential centrifugation by sedimentation coefficient to remove cells, organelles and microvesicles; ≥100,000 × g to pellet small EVs	Laboratory “gold standard”; no chemical reagents; medium-to-high purity EVs from conditioned media	Expensive and labor-intensive; high g-force causes EV deformation/aggregation; heavy co-pelleting of proteins and lipoproteins from biofluids	Conventional preparation of EVs from MSC-conditioned media; basic research aligned with historical UC-based literature	Difficult to run continuously or under GMP; largely replaced by TFF-based processes for clinical manufacturing
Density-gradient ultracentrifugation (Jia et al., 2022)	Sucrose or iodixanol gradients separate EVs and contaminants according to buoyant density	Very high purity; efficient removal of protein/lipoprotein contaminants; allows density-defined EV subfractions	Gradient preparation is complex; long run time and low recovery; unsuitable for high-throughput processing	High-purity omics; studies of EV heterogeneity and density subpopulations	Best used as an analytical or polishing step rather than a primary large-scale manufacturing method
Ultrafiltration (UF) (Li B et al., 2025)	Size-based retention of EVs on MWCO membranes; often combined with SEC or UC	Rapid; handles large volumes; no polymers added; easily integrated with other methods	Membrane clogging and adsorption cause losses; shear stress may damage EVs; soluble proteins are still co-concentrated	Pre-concentration of conditioned media or biofluids; UF–SEC workflows	Technically scalable but mainly used as small-scale or auxiliary step preceding TFF
Tangential-flow filtration (TFF) (Li M et al., 2025)	Cross-flow filtration enabling continuous concentration and diafiltration while reducing membrane fouling	Closed system compatible with GMP; easily scaled from liters to hundreds of liters; gentle on EV structure and function	Capital and consumable costs are high; usually requires SEC/AEC polishing for high purity	Core technology for manufacturing clinical-grade MSC-EV products	Strong advantages in scale-up and standardization; one of the most promising technologies for industrialization
Size-exclusion chromatography (SEC) (Kim et al., 2025)	Porous resins separate particles according to hydrodynamic volume; EVs elute before small proteins	Mild conditions preserve EV integrity; highly reproducible; compatible with many sample types	Limited loading volume and dilute eluates requiring reconcentration; modest resolution from lipoproteins	Purification of EVs from plasma, urine and other biofluids; polishing after UF/TFF; sample prep for omics	Parallel or serial columns allow moderate scale-up, making SEC suitable as a GMP polishing step
Anion-exchange chromatography (AEC) (Saari et al., 2023)	Negatively charged EV membranes bind to positively charged resins and are eluted by salt gradients	High-purity polishing; easily combined with TFF/SEC for two-dimensional purification; amenable to continuous processing	Requires fine optimization of buffer and ionic strength; high-salt eluates must be thoroughly desalted; sensitive to charge heterogeneity between EV sources	Final purification of clinical-grade EV products; removal of charged protein and nucleic-acid contaminants	Highly compatible with TFF and attractive as a module in industrial EV workflows
Polymer precipitation (PEG and related) (Huang et al., 2025)	Volume-exclusion effects of polymers cause EVs to precipitate at low-speed centrifugation	Very simple and equipment-light; apparently high recovery; useful for crude enrichment and early screening	Co-precipitates large amounts of proteins, lipoproteins and immune complexes; low purity; residual PEG may interfere with assays; usually requires subsequent SEC/UC	Exploratory studies or enrichment of small clinical samples followed by further purification	Not recommended for direct manufacture of clinical-grade products; mainly for early-stage screening
Immunoaffinity/immunomagnetic capture (Ljungström and Oltra, 2025)	Antibodies or aptamers against CD9/CD63/CD81 or cell/disease-specific markers capture EVs on solid supports	Very high specificity and purity; enriches EVs from defined sources or subpopulations; works with very small sample volumes	Antibodies and beads are costly; only a subset of EVs is captured; harsh elution conditions may damage EVs	Liquid-biopsy assays; diagnostic and mechanistic studies of defined MSC-EV subpopulations	Limited scalability; better suited as diagnostic/analytical module than production line
Microfluidic chips (Wu Y et al., 2022)	Separate EVs in microscale channels using physical (size, acoustics, inertia) or biochemical (immunoaffinity) mechanisms	Very low sample requirement; rapid analysis; easy integration with downstream detection for “sample-in, answer-out” systems	Devices and fabrication not yet fully standardized; throughput is low; most systems remain prototypes	On-chip EV isolation and point-of-care testing from small-volume biofluids; high-throughput screening	Attractive as diagnostic devices and online monitoring/sorting modules in production processes
EXODUS and related automated ultrafast systems (Chen et al., 2021)	Negative-pressure oscillation plus dual-frequency vibration/ultrasonic nano-filtration rapidly trap and release EVs on nanoporous membranes	Very fast with high recovery and purity; high degree of automation and reproducibility; compatible with ∼10 μL to several hundred mL of sample	Instruments and consumables are expensive; highly viscous samples still require pre-treatment; currently used mainly in diagnostic and research settings	Discovery and validation of EV biomarkers from diverse body fluids; high-sensitivity EV detection	Strong potential for adoption in clinical laboratories as diagnostic/quality-control platforms, though not yet mainstream for production
AF4 and other field-flow fractionation (Zhang et al., 2018)	Cross-flow field and Brownian diffusion in an unpacked channel separate particles by hydrodynamic size/density	Gentle separation with high resolution; finely resolves EV subpopulations and exomeres; readily coupled to MALS/NTA/MS	Instruments are costly and method development complex; throughput is limited, so mainly analytical rather than preparative	High-resolution studies of EV heterogeneity; advanced characterization of particle size and impurities in clinical-grade products	Highly valuable as analytical and quality-control tools, but not well suited as primary preparative methods

EV, extracellular vesicle; MSC, mesenchymal stromal/stem cell; dUC, differential ultracentrifugation; UC, ultracentrifugation; UF, ultrafiltration; MWCO, molecular weight cut-off; SEC, size-exclusion chromatography; TFF, tangential-flow filtration; AEC, anion-exchange chromatography; PEG, polyethylene glycol; AF4, asymmetric flow field-flow fractionation; MALS, multi-angle light scattering; NTA, nanoparticle tracking analysis; MS, mass spectrometry; GMP, Good Manufacturing Practice; EXODUS, an automated ultrafast EV isolation platform.

#### 6.1.2 Quality control: from “this is an EV” to “a controllable MSC-EV drug product”

MSC-derived EVs are inherently complex and heterogeneous biological products, posing major challenges for QC and standardization. Most preclinical and early clinical studies still follow the minimal characterization framework advocated by MISEV 2018/2023: particle size distribution (NTA/TRPS), morphology (TEM/AFM), a limited set of positive markers (CD9/CD63/CD81 plus TSG101/ALIX) and negative markers (e.g., APOA1, GM130), alongside impurity metrics such as total protein, residual DNA, or lipoprotein contamination to define purity (Welsh et al., 2024). This framework improves comparability, but it largely answers “Is this an EV?” rather than “Is this an MSC-EV product with reproducible pharmacology?”

For MSC-EVs intended as regenerative therapeutics, “EV identity” is necessary but far from sufficient. MSC source, culture conditions, and isolation processes can profoundly reshape cargo composition and functional programs, producing markedly different immunomodulatory, osteogenic/chondrogenic, and pro-angiogenic effects—an issue repeatedly emphasized in recent MSC-EV therapeutic and engineering reviews (Almeria et al., 2022). Therefore, a translational MSC-EV product requires a multidimensional “identity” definition: not merely “EV-ness,” but a formulation derived from a defined MSC population with reproducible molecular and functional phenotypes. At minimum, this identity should incorporate three interconnected layers:

1. Producer-cell attributes: MSC tissue origin (bone marrow, umbilical cord, adipose, etc.), donor background, immunophenotype (CD73/CD90/CD105^+^; CD34/CD45^-^), passage number, and karyotypic stability—upstream variables that shape secretion programs and safety.
2. EV surface/intraluminal signatures: beyond classical markers, “fingerprint-like” profiling of MSC-EV–enriched molecules (e.g., CD44, CD146, integrin subunits) and tissue-origin–associated proteins/glycoforms, supported by quantitative proteomic/lipidomic features to distinguish MSC sources or process-dependent products, consistent with emerging evidence on EV heterogeneity and functional divergence in tissue repair.
3. Cargo profiles and pathway modules linked to mechanism of action (MoA): miRNA-seq/RNA-seq/proteomics to define bone/cartilage repair–relevant modules (immune regulation, osteogenesis, angiogenesis), and to relate these molecular features to standardized *in vitro* readouts—thereby binding “identity” to an expected MoA.

Crucially, EV heterogeneity should be treated as a defining property rather than averaged away. High-impact reviews have highlighted that even within a single MSC-EV preparation, EV subpopulations stratified by size, density, or surface-marker combinations may exert distinct—sometimes opposing—effects on repair, immune regulation, or adverse outcomes; producer-cell state and isolation workflows systematically reshape these subpopulation compositions (Van De Wakker et al., 2023). Consequently, “MSC-EV identity” is best viewed as a composite of critical quality attributes (CQAs): establishing an operable window across size distribution, producer-cell features, EV subpopulation structure, and functional cargo that both predicts efficacy and can be robustly controlled under GMP (Giebel and Lim, 2025). Recent CQA-focused commentaries on MSC-sEVs argue that without this multidimensional CQA-based identity, MSC-EVs are unlikely to be regulated as truly controllable biologic drug products.

Operationalizing the three-layer identity framework in clinical quality control requires mapping each layer to measurable critical quality attributes (CQAs) and implementing them as a tiered control strategy spanning upstream producer-cell qualification, downstream EV physicochemical identity/purity testing, and MoA-linked cargo/potency verification (Xu et al., 2025). In practice, producer-cell release tests may include MSC immunophenotyping (CD73/CD90/CD105^+^; CD34/CD45^-^), viability, passage/senescence status, karyotypic stability, and microbiological safety (e.g., sterility and *mycoplasma*) prior to conditioned-medium harvest (Dominici et al., 2006). EV-level CQAs can then be assessed by orthogonal methods such as particle concentration/size distribution (NTA/TRPS), morphology (TEM), positive/negative marker panels (e.g., CD9/CD63/CD81, TSG101/ALIX; APOA1, GM130), and impurity metrics (e.g., protein-to-particle ratio and residual DNA/lipoproteins), with acceptance windows defined relative to a qualified reference lot and controlled under GMP(Théry et al., 2018). Finally, MoA-linked cargo modules (e.g., angiogenesis-, immunomodulation-, and osteogenesis-related miRNAs/proteins) can be monitored by targeted qPCR/ELISA panels and integrated with functional potency assays (Section 6.1.3) to support batch release and comparability after process changes (Table 4).

**TABLE 4:** Operationalizing the three-layer identity criteria into clinical QC: suggested CQAs and example assays for MSC-EV products intended for vascularized bone regeneration.

QC dimension	Representative CQAs	Example assays/Metrics	Implementation in clinical QC
Producer-cell attributes	MSC tissue origin/donor eligibility; immunophenotype; passage/senescence; genomic stability; microbiological safety	Donor screening; flow cytometry (CD73/CD90/CD105^+^; CD34/CD45^-^); viability; karyotype/CGH; sterility & *mycoplasma* testing	Upstream qualification (master/working cell bank release) and in-process controls prior to conditioned-medium harvest
EV surface/intraluminal signatures	Particle concentration & size distribution; morphology; EV marker panel; purity/impurities; formulation attributes	NTA/TRPS; TEM; CD9/CD63/CD81, TSG101/ALIX (WB/flow); negative markers (e.g., APOA1, GM130); protein-to-particle ratio; residual DNA/endotoxin	In-process and final drug-product identity/purity release testing; stability monitoring
MoA-linked cargo modules & potency	Angio-/osteo-/immunomodulatory miRNA/protein modules; functional bioactivity normalized to particle dose	Targeted qPCR/ELISA panels; endothelial migration/tube formation; macrophage polarization; ALP/mineralization assays	Batch-release potency and comparability after process changes; acceptance ranges defined vs. a qualified reference lot

#### 6.1.3 The missing potency assay framework

Building on the three-layer CQA-based identity framework described above (Section 6.1.2; Table 5), potency assays provide the functional layer of clinical QC by translating molecular identity into predictable biological activity. Compared with morphological and molecular characterization, MSC-EVs remain even more underdeveloped in potency testing. Multiple systematic reviews note that most preclinical and early clinical studies still rely on an empirical paradigm: dosing by protein amount or particle number and then observing animal or clinical endpoints, with relatively few regulator-acceptable, reproducible *in vitro* assays that quantitatively measure product potency. A landmark 2021 review by Gimona and colleagues on behalf of the ISCT Exosomes Committee emphasized that potency assays for MSC-sEVs must be MoA-centered, provide quantitative readouts, and sensitively reflect changes in CQAs; otherwise, they cannot support scientifically grounded dose-setting, batch release, or comparability across process changes (Gimona et al., 2021). In other words, even if GMP scale-up is achieved, without a robust potency framework MSC-EVs remain closer to “research material” than a “regulatable therapeutic.”

**TABLE 5:** Clinical trials of MSC-EVs in bone regeneration.

NCT ID	Indication	EV source	Route	Phase	Primary endpoint(s)	Status/Last update posted
NCT05520125	Segmental long-bone defect/fracture with bone loss	Autologous or allogeneic MSCs enriched with their own extracellular vesicles (MSC + EV cell product)	Surgical implantation into defect (with internal fixation ± bone grafting)	Phase 1/2	Proportion of complete reconstruction at 12 months; treatment-related adverse events (focus on first month)	Not yet recruiting/Unknown; 2022-08-29
NCT04998058	Alveolar bone atrophy/loss requiring bone grafting (e.g., sinus augmentation)	Autologous adipose-derived MSC conditioned medium/secretome (EV-rich)	Local application mixed with synthetic bone substitute during grafting	Phase 1/2	Change in bone density and quantity (baseline, 90 and 180 days); bone quantity at 180 days	Not yet recruiting; 2025-06-05
NCT04270006	Moderate-to-severe periodontitis with alveolar bone loss	Autologous adipose-derived stem cell exosomes (ADSC-Exos)	Local injection into periodontal pockets after scaling/root planing	Early Phase 1	Changes in gingival inflammation, probing depth (PD), clinical attachment level (CAL), and bone level	Unknown; 2020-02-17

Registry information may be incomplete and subject to change (e.g., MSC tissue source, EV dose units, and dosing schedule). Abbreviations: NCT, ClinicalTrials.gov identifier; MSC, mesenchymal stromal/stem cell; EV, extracellular vesicle; ADSC, adipose-derived stem/stromal cell; PD, probing depth; CAL, clinical attachment level.

From the perspective of bone and osteochondral repair, an ideal MSC-EV potency panel should map onto core MoAs in regeneration and span at least three complementary dimensions:

Osteogenic/chondrogenic activity: standardized assays in MSCs or osteogenic/chondrogenic cell lines that quantify ALP activity, mineralized nodule formation, and expression of cartilage/bone markers (e.g., type II collagen, aggrecan), reflecting EV support for matrix formation and phenotype maintenance.

1. Immunomodulation and microenvironmental reprogramming: readouts such as macrophage M1→M2 polarization, T-cell proliferation and Th1/Th17–Treg balance, and osteoclastogenesis, capturing EV effects on inflammation–repair balance and remodeling niches.
2. Angiogenesis and matrix remodeling: endothelial migration/tube formation assays, and expression of MMPs/TIMPs and related remodeling signals, reflecting EV actions in building a permissive “scaffold microenvironment.”
3. Recent commentaries on MSC secretome/EVs in regenerative medicine have suggested borrowing concepts from biologics and cell therapy guidance—using such functional readouts as candidate potency metrics and then narrowing to one or two core assays most predictive of *in vivo* efficacy.

Encouragingly, several studies have begun operationalizing these concepts. Kaur et al. established a bioassay in experimental autoimmune uveitis to quantify MSC-EV suppression of Th1/Th17 responses and proposed standardized particle-normalized TGF-β1 levels and inhibition of T-cell activation pathways as surrogate potency markers predicting *in vivo* disease amelioration (Kaur et al., 2024). Separately, Adamo et al. introduced the DetectEV enzymatic assay, quantifying luminal enzyme activity together with membrane integrity to translate EV bioactivity into calibratable enzymatic units. This system has been shown to discriminate functional differences across EV sources and processes in multiple cellular models and has been proposed as a candidate “general-purpose” QC tool (Adamo et al., 2025). While such methods still require further validation and standardization, they converge on a central point: without MoA-aligned, quantitative potency assays, it is not feasible to define dose equivalence, batch release criteria, or process comparability. Conversely, once potency is anchored to CQAs and MoA, MSC-EVs can more credibly advance into high-quality clinical trials and regulatory pathways for bone repair.

### 6.2 Available clinical evidence and translational outlook

Although substantial challenges remain for applying MSC-derived extracellular vesicles (MSC-EVs) to bone regeneration, the translational outlook remains compelling. At present, clinical evidence for MSC-EVs in skeletal repair is still early and sparse: large, adequately powered trials are lacking, and EV-based products for bone regeneration remain investigational rather than established clinical therapies.

Consistent with this gap, clinical-trial registries contain hundreds of EV-related entries overall, but only a small fraction directly address bone defects or fracture repair. For example, one review reported 292 EV-related trials registered on ClinicalTrials.gov as of January 2025 (using “extracellular vesicle”/“exosome” keywords), underscoring rapid field expansion but also heterogeneity in indications. Another analysis identified 383 registered trials using broad EV-related search terms, yet only 60 unique trials used EVs as the primary therapeutic intervention, highlighting that many registrations focus on biomarkers or secondary endpoints rather than EV therapeutics *per se*. Against this broader landscape, trials explicitly targeting bone defects remain uncommon.

Despite the limited trial volume, early studies and case reports provide preliminary signals for feasibility and safety, supporting continued development of MSC-EV–based strategies for bone regeneration (Table 5).

#### 6.2.1 Available clinical evidence

##### 6.2.1.1 Segmental bone defect repair

To date, the only registered clinical study that has applied a stem cell–exosome–related product directly in patients with bone tissue defects is NCT05520125, conducted in Belarus (“Treatment of Patients with Bone Tissue Defects Using Mesenchymal Stem Cells Enriched by Extracellular Vesicles”). Registry summaries describe it as an early-phase, interventional, parallel-assignment study, and third-party trackers commonly label it as Phase 1.

The investigational intervention is a MSC product enriched with its own extracellular vesicles, prepared by first establishing a small-EV/exosome production workflow and then co-formulating MSCs with their autologous EVs into a combined cellular product (“mesenchymal stem cells enriched by their own extracellular vesicles”). The control arm receives standard management of segmental bone defects (e.g., conventional bone grafting and internal fixation). In the experimental arm, the MSC + EV product is implanted into the segmental defect site in conjunction with metal internal fixation and/or bone grafting; however, detailed EV parameters (e.g., particle number vs. protein dose), dosing frequency, and rules for repeat implantation have not been publicly disclosed.

The primary endpoints include: (i) the proportion of patients achieving complete reconstruction of segmental bone defects at 1 year, reflecting restoration of bony continuity and clinical union; and (ii) the incidence and severity of treatment-related adverse events, with particular attention to early safety within the first month after treatment. Secondary endpoints focus on imaging-based measures of union quality and functional recovery (e.g., limb weight-bearing capacity and pain scores).

As of 8 January 2026, publicly available registry trackers still list the study with a “last known” recruitment status of not yet recruiting/unknown, and no peer-reviewed efficacy or safety results have been published. Nonetheless, this study represents an important first step: by benchmarking an MSC-EV–enriched product against standard care and adopting complete bony reconstruction plus safety as core outcomes, it provides a pragmatic template for future multicenter randomized trials in population definition, endpoint selection, and regulatory dialogue.

##### 6.2.1.2 Alveolar bone defect repair and periodontal regeneration

Katagiri et al. (2016) reported the first exploratory human study testing an MSC-derived secretome approach for alveolar bone regeneration (Katagiri et al., 2016). Eight patients requiring bone augmentation prior to dental implant placement received β-tricalcium phosphate (β-TCP) or collagen scaffolds soaked with bone marrow MSC-conditioned medium (MSC-CM)—a complex mixture containing exosomes/EVs and other soluble factors—followed by implantation into alveolar defects. No marked systemic or local adverse reactions were observed. Imaging suggested early new bone formation across cases, and biopsies in selected patients confirmed newly formed bone with minimal inflammatory cell infiltration, supporting the feasibility and apparent safety of MSC-CM (including EV-containing fractions) for alveolar bone regeneration in a Phase 1 setting.

Direct EV-based periodontal regeneration trials remain limited. NCT04270006 is currently the only registered clinical study explicitly evaluating stem cell–derived exosomes for periodontitis/periodontal regeneration. Registry summaries describe an early-phase, single-arm, open-label design in which, after conventional supragingival/subgingival debridement and root planing (SRP), patients with moderate-to-severe periodontitis and evident alveolar bone resorption receive local administration of autologous adipose-derived MSC exosomes (ADSC-Exos) prepared from each participant’s own adipose tissue–derived ADSCs. Primary outcomes include probing depth (PD), clinical attachment level (CAL), and gingival inflammation indices, complemented by cone-beam CT metrics (alveolar bone height and bone density) to evaluate whether ADSC-Exos can augment SRP by both controlling inflammation and promoting attachment gain and bone regeneration. To date, only the study protocol has been posted, and no formal results have been released.

In addition, NCT04998058 is a Phase 1 study in Brazil evaluating autologous MSC culture-derived products (conditioned medium/secretome, described as rich in signaling molecules including EVs) as enhancers of bone formation in bone grafting, with imaging-based assessments of graft bone formation (e.g., density, volume, and maturation). As of late 2025, third-party trackers list the study as not yet recruiting, with a posted date in December 2025, and peer-reviewed outcomes have not yet appeared.

#### 6.2.2 Translational outlook

Overall, the clinical translation of mesenchymal stromal/stem cell–derived extracellular vesicles (MSC-EVs) for bone regeneration remains at an early stage, yet the field has gained clear momentum. Across currently available human studies, MSC-EV–based interventions have generally shown favorable tolerability, with few serious adverse events reported, supporting the notion that MSC-EVs can be administered with an acceptable early safety profile. On the efficacy side, several Phase I studies in indications such as alveolar bone augmentation and osteoarthritis have reported trends toward accelerated tissue repair or symptom relief. However, rigorous randomized controlled data remain limited. Notably, at least one well-designed randomized controlled trial (RCT) in knee osteoarthritis did not demonstrate a statistically significant benefit of MSC-EVs over placebo, underscoring that early signals from open-label studies do not necessarily translate into definitive efficacy.

Accordingly, routine clinical adoption of MSC-EVs in orthopedics will require progress on several fronts: (i) adequately powered controlled trials to confirm efficacy; (ii) standardized, traceable manufacturing and quality-control systems to ensure batch-to-batch reproducibility; (iii) rational optimization of dose, dosing units, and dosing frequency; and (iv) deeper mechanistic clarification to define the best-fit indications and responder populations.

From a global perspective, the number of registered MSC-EV–related clinical studies continue to rise. Beyond current work in alveolar bone augmentation and osteoarticular diseases, plausible next targets include osteoporosis (e.g., systemic delivery to improve whole-body bone mass), refractory nonunion, and large/segmental bone defects. Translational efficiency may further improve by integrating MSC-EVs with smart biomaterials (injectable hydrogels, 3D-printed scaffolds) to enable targeted delivery and controlled release, or by preconditioning donor cells (hypoxia, pharmacologic stimulation) to potentiate osteo-angiogenic activity. Finally, long-term follow-up studies remain indispensable to define durability, stability, and broader effects on the bone microenvironment, particularly in contexts requiring repeated administration.

## 7 Future directions

Building on the evidence-based translational outlook in Section 6.2.2, this section summarizes broader future directions for MSC-EV–mediated vascularized bone regeneration, spanning mechanistic dissection, engineering and delivery optimization, and translational standardization.

Mechanistic priority—phase- and cell-state–resolved angiogenesis–osteogenesis coupling in bone repair. A key bone-specific gap is to define how MSC-EVs regulate the coordinated actions of endothelial subtypes, immune cells (especially macrophage states), osteoprogenitors, and perivascular support cells across inflammation, callus formation, mineralization, and remodeling. Although EV-borne miRNAs/proteins are frequently linked to endothelial activation and osteogenic differentiation, the coupling network in bone—including spatiotemporal dynamics and context-dependent feedback under endochondral versus intramembranous repair—remains incompletely mapped. Future work integrating spatial multi-omics with hypothesis-driven bone defect models is needed to reconstruct causal circuitry that connects EV exposure to vascular remodeling and subsequent bone formation.

Engineering priority—bone-focused targeting and potency enhancement. Engineering strategies (preconditioning, genetic modification, and surface functionalization) should be evaluated with explicit orthopaedic goals: increasing lesion-site enrichment (e.g., bone-homing or injured endothelium–targeting designs), reinforcing osteo-angiogenic coupling rather than isolated angiogenesis, and minimizing risks such as aberrant neovascularization. Given heterogeneity across MSC sources and culture conditions, bone-indication development will benefit from standardized, reproducible engineering pipelines that are benchmarked using bone-relevant potency readouts (e.g., coordinated endothelial–osteoprogenitor responses) rather than generic assays alone.

Delivery priority—defect-site exposure, retention, and “combination-product” readiness. Because systemic half-life is short and intrinsic targeting is limited, orthopaedic translation often depends on local delivery formats that prolong retention in defects or joints—particularly EV–biomaterial depots (injectable hydrogels, porous scaffolds) that can be integrated into clinically familiar reconstruction workflows. A central bone-specific challenge is matching release kinetics to the tempo of vascularized bone repair and to the mechanical environment, while preserving EV bioactivity during scaffold fabrication and sterilization. Mechanistic studies on EV–biomaterial synergy should be designed to explain why certain depots enhance coupling, enabling rational “stage-aware” delivery rather than continuous, non-specific pro-angiogenic stimulation.

*In vivo* fate and exposure–response—quantitative frameworks in mineralized tissues. Establishing biodistribution, defect-site residence time, clearance routes, and immune interactions remains necessary, but in bone this question has additional layers: lesion accessibility within mineralized matrices, marrow–vascular niche interactions, and the need to correlate EV retention with perfusion restoration and structural union. Future studies should therefore incorporate quantitative tracing and imaging that are feasible in bone settings and relate exposure metrics to vascular indices and bone outcomes.

Safety and durability—orthopaedic-specific concerns. Beyond general immunogenicity and repeated-dosing considerations, orthopaedic applications warrant focused assessment of risks relevant to bone/joint microenvironments, including ectopic calcification or maladaptive remodeling, undesired vascular architectures, and potential attenuation of benefit over prolonged follow-up—particularly in scenarios requiring repeated administration.

Clinical development—indication selection and endpoint rigor. Consistent with the limitations summarized in the translational outlook section, the evidence base remains constrained by the scarcity of large, well-controlled trials. For bone regeneration indications, translational progression should prioritize (i) large-animal models that capture human-like biomechanics, fixation contexts, and defect complexity, and (ii) multicenter, adequately powered RCTs with orthopaedically meaningful structural and functional endpoints (e.g., CT-based bridging/union, time to union, reoperation rates, and validated functional recovery metrics), rather than relying primarily on subjective outcomes.”

## 8 Conclusion

MSC-EV–based bone regeneration strategies show substantial potential to promote angiogenesis and osteogenic repair, offering a compelling cell-free therapeutic paradigm for bone defects. While significant uncertainties remain—particularly in manufacturing standardization, delivery optimization, and high-quality clinical validation—progress is accelerating. With continued resolution of these key barriers and rigorous confirmation of safety and efficacy through well-designed clinical trials, MSC-EV–mediated bone regeneration is positioned to move from experimental studies toward real-world clinical implementation, ultimately delivering tangible benefits for patients with complex bone defects and osteoarticular injury.

## Glossary

ADSC: adipose-derived stromal cell
AF4: asymmetric flow field-flow fractionation
AFM: atomic force microscopy
AEX: anion-exchange chromatography
AEC: anion-exchange chromatography
ALN: alendronate
BMP2: bone morphogenetic protein 2
BMD: bone mineral density
BMSC: bone marrow mesenchymal stromal/stem cell
BV/TV: bone volume/total volume
CAL: clinical attachment level
CHA: coral acid hydroxyapatite
CMC: chemistry, manufacturing, and controls
CQA: critical quality attribute
DF-PEG: dysfunctional polyethylene glycol
dUC: differential ultracentrifugation
ECM: extracellular matrix
EPC: endothelial progenitor cell
EV: extracellular vesicle
GMP: Good Manufacturing Practice
GCS: glycol chitosan
HIF-1α: hypoxia-inducible factor 1α
HSC: hematopoietic stem cell
HUVEC: human umbilical vein endothelial cell
IHC: immunohistochemistry
IKDC: International Knee Documentation Committee
KL: Kellgren–Lawrence
KOOS: Knee injury and Osteoarthritis Outcome Score
MALS: multi-angle light scattering
miRNA: microRNA
MOF: metal–organic framework
MoA: mechanism of action
MPN: metal–polyphenol network
MPS: mononuclear phagocyte system
MRI: magnetic resonance imaging
MSC: mesenchymal stem/stromal cell
MWCO: molecular weight cutoff
NTA: nanoparticle tracking analysis
NR: not reported
OA: osteoarthritis
OCN: osteocalcin
PBS: phosphate-buffered saline
PD: probing depth
PCL: polycaprolactone
PEG: polyethylene glycol
PK: pharmacokinetics
PLA: polylactic acid
sEV: small extracellular vesicle
SEC: size-exclusion chromatography
SF: silk fibroin
SHED: stem cells from human exfoliated deciduous teeth
Tb.N: trabecular number
TEM: transmission electron microscopy
TFF: tangential-flow filtration
TRPS: tunable resistive pulse sensing
UF: ultrafiltration
VAS: visual analog scale
VEGF: vascular endothelial growth factor
WOMAC: Western Ontario and McMaster Universities Osteoarthritis Index
β-TCP: beta-tricalcium phosphate
